# Extracellular Vesicles from the Myocyte Secretome Contribute In Vitro to Creating an Unfavourable Environment for Migrating Lung Carcinoma Cells

**DOI:** 10.3390/biology14111578

**Published:** 2025-11-11

**Authors:** Dona Mannaperuma, Dan Stratton, Sigrun Lange, Jameel M. Inal

**Affiliations:** 1 School of Life Sciences, Cellular and Molecular Immunology Research Centre, London Metropolitan University, 166-220 Holloway Road, London N7 8DB, UK; dm19aab@herts.ac.uk (D.M.); dan.stratton@open.ac.uk (D.S.); 2 Biosciences Research Group, Extracellular Vesicles Research Unit, School of Health, Medicine and Life Sciences, University of Hertfordshire, Hatfield AL10 9AB, UK; 3 School of Life, Health & Chemical Sciences, The Open University, Milton Keynes MK7 6AA, UK; 4 Pathobiology and Extracellular Vesicles Research Group, School of Life Sciences, University of Westminster, 115 New Cavendish Street, London W1W 6UW, UK; s.lange@westminster.ac.uk; 5 School of Human Sciences, Cell Communication in Disease Pathology, London Metropolitan University, 166-220 Holloway Road, London N7 8DB, UK

**Keywords:** extracellular vesicles (EVs), EV-transplant, lung cancer, metastasis, skeletal muscle, mitochondria, lysosomes, apoptosis, decorin

## Abstract

Cancer progression in skeletal muscle (SkM) is very rare, and mechanisms remain unclear. This study assessed the potential of SkM (myocyte)-derived EVs (C2C12-EVs) as anti-cancer agents. Using murine in vitro models, we showed that following treatment with C2C12-EVs, lung carcinoma cells failed to colonise SkM cells, and that C2C12-EVs selectively exerted apoptosis on cancer cells. Uptake of C2C12-EVs by carcinoma cells caused changes in lysosomal function and mitochondrial membrane properties inducing cell death with elevated caspase 3 and 9. The C2C12-EVs also inhibited cell proliferation, affecting cell cycle arrest at S phase and inhibited cell migration. Proteomic analysis of C2C12-EV cargoes highlighted functional enrichment pathways involved in lysozyme function, HIF-1 and PI3K-Akt signalling, regulation of actin cytoskeleton, pyruvate metabolism, platelet activation, and protein processing in ER. Decorin, a muscle cell-specific cytokine released from myocytes in response to stress, was significantly enriched in C2C12-EVs and may contribute to C2C12-EVs’ inhibitory activity on cancer cells. C2C12-EVs may suppress cancer and potentially be used as therapeutic agents for cancer metastasis.

## Simple Summary

Skeletal muscle is rarely affected by cancer, which indicates that these muscle cells contain cancer-resistant properties. Cells release small membrane vesicles, extracellular vesicles (EVs), which are important for communication between cells, and may be protective if released from certain cells, but can also contribute to disease signalling, including in cancer. In this study, we isolated EVs from skeletal muscle cells and applied them to lung cancer cells. We found that the skeletal muscle EVs have anti-cancer signatures and stopped the cancer cells from spreading and caused them to die. Our findings highlight the potential of skeletal muscle EVs as anti-cancer agents and the importance of skeletal muscle and their EVs in healthy ageing and to protect human health in conditions when skeletal muscle mass is compromised.

## 1. Introduction

Extracellular vesicles (EVs) play important roles in cellular communication in both normal physiology and in disease processes. EVs are released from most cell types and carry cell-specific macromolecular cargo including proteins, lipids, nucleic acids and non-coding RNAs (miRNA and lncRNA), mediating intercellular communication and altering the status of recipient cells [1,2,3]. EVs have multifaceted roles in modulating immune responses to promote cancer, for example promoting cancer metastasis and tumour growth [4,5,6]. Interestingly though, some cell-derived EVs can decrease tumour growth, as, for example, observed in mice, where EVs from dendritic cells containing α-fetoprotein induced robust immune responses that suppressed tumour growth, or where EVs induced cytotoxic T cell responses and increased activation [7,8,9,10]. EVs have also been described as agents of cell repair and homeostasis, with reports of platelet EVs, for example, being able to repair myocardial injury after an infarction [11], and mesenchymal stem cell EVs promoting tissue repair in various organs including in heart, bone, skin, lung, kidney, liver and the central nervous system [12,13,14,15,16,17]. EVs, including engineered ones, are furthermore useful as carriers of pharmacological and genetic therapeutic agents [18,19,20,21]. Given such diverse roles of EVs in physiological and pathobiological processes, it is important to further understand how EVs from different cell types affect cancer growth and metastasis, as well as any protective and anti-cancer properties of certain cell-type-specific EVs.

Importantly, while cancer metastasis occurs in most organs and tissues, it is rare in skeletal muscle [22,23,24,25], with a prevalence varying from 0.03 to 5.6% in autopsy series [26,27,28] and 1.2–1.8% in radiological series [29,30], mostly from primary lung carcinomas and malignant melanomas. Given that skeletal muscle is a highly vascularised tissue comprising about 50% of body mass, the rarity of metastasis to skeletal muscle is surprising. As physical activity negatively regulates tumour cell growth in skeletal muscle [31], it has been postulated that this might be due to the high levels of lactic acid [31] or the influence of β-adrenergic receptors on the variability of blood flow between a resting and exercise state [31]. In other studies, conditioned media from skeletal muscle were shown to be cytostatic to tumour cells [32,33] and to inhibit metastatic cells and tumour growth, possibly due to low molecular weight factors such as adenosine [34]. While EVs have been identified to be modulated in response to exercise [35], and the link between exercise and cancer protection has been highlighted [31,36], current understanding of the possible relationship between muscle-derived EVs and protection from cancers remains limited.

Myokines are skeletal muscle cell-specific cytokines released from myocytes in response to stress, including exercise. There are over 600 myokines in human myocyte-conditioned medium, including myostatin, IL-6, IL-15, irisin, myonectin, decorin, SPARC and FSTL-1, some of which are involved in cancer suppression. Irisin, oncostatin and SPARC have, for example, been shown to suppress colon and breast cancer growth [37], and IL-6 and SPARC play roles in the prevention of cancers by exercise [38]. Roles for decorin were reported via TGF-β modulation, showing inhibition of cancer growth, survival, metastasis and angiogenesis [39]. Decorin overexpression was furthermore shown to induce apoptosis and cell cycle arrest in rat mesangial cells [40], and in a decorin knockout mouse model, 30% of mice developed spontaneous intestinal tumours and invasion [41]. While EVs have been identified as delivery vehicles for some myokines [42], EV-mediated transport of decorin has not been assessed to date but may play roles in cellular mediation in cancer communication as well as in anti-cancer processes.

Although most research to date has focused on the pro-tumourigenic properties of EVs, the increasing evidence of some EVs’ anti-tumourigenic effects may be exploited for therapeutic benefits. Studies have, for example, shown that EVs carrying miR-375 inhibit colon cancer cell proliferation and invasion by blocking Bcl-2 [43], while EVs carrying circulating RNA-0051443 inhibited hepatocellular carcinoma progression by inducing apoptosis [44]. Mesenchymal stem cells containing miRNA-100 were furthermore shown to inhibit breast cancer angiogenesis [45]. This points to roles for various EV cargoes in anti-cancer processes. Whilst RNA species have been assessed, overall, there has been less attention paid to proteomic cargoes. Given the potential anti-tumourigenic properties of skeletal muscle-derived EVs, this current study assessed the effects of myocyte-derived EVs on lung carcinoma cells. For this purpose, we compared anti-cancer effects of skeletal muscle-derived EVs, isolated from a murine myogenic myocyte cell line (C2C12), with those from a non-myogenic murine fibroblast (“control”) cell line (NIH-3T3), and assessed effects on the tumourigenic and metastatic murine lung carcinoma cell line (CMT 64/61).

The aim of this study was therefore to (i) isolate and characterise EVs from murine myocytes (C2C12)—with a focus on proteomic cargoes; (ii) assess the effects of the myocyte-derived EVs on cell proliferation, cell migration, cytotoxicity and apoptosis of metastatic lung carcinoma cells (CMT 64/61); and (iii) identify candidate bioactive protein(s) transported in the myocyte EVs that could be involved in preventing cancer metastasis and cancer progression in the skeletal system.

## 2. Materials and Methods

### 2.1. Cell Cultures

Adherent murine myoblast cells (C2C12; ECACC No. 91031101, Salisbury, UK) and murine fibroblast cells (NIH-3T3; ECACC No. 93061524) were maintained in Dulbecco’s modified Eagle’s medium (DMEM; Sigma-Aldrich, Gillingham, UK) supplemented with EV-depleted 10% or 2% (*v*/*v*) Foetal Bovine Serum (FBS; Sigma-Aldrich), 2 mM Glutamine and 1% penicillin-streptomycin (Sigma-Aldrich) at 37 °C, 5% CO_2_. Murine lung carcinoma cells (CMT 64/61; Ximbio No. 152751, London, UK) were maintained in Waymouth’s MB 752/1 medium (WM) (Sigma-Aldrich) supplemented with 10% FBS and 1% penicillin-streptomycin (Sigma-Aldrich). They were adapted to DMEM by changing, upon each passage, to 90% WM + 10% DMEM, then 75% WM + 25% DMEM, until in 100% DMEM. All cells were split at 70% confluency using standard procedures. Throughout all experiments, the cells used were within 10 passages. Cells were regularly checked for contamination by PCR to detect mycoplasma 16S rRNA (LookOut^®^ Mycoplasma PCR Detection Kit, Sigma-Aldrich).

#### Promotion of C2C12 Cell Differentiation

The myoblast stage cells (C2C12) were differentiated into myocytes once at 90% confluence by washing twice in PBS and replacing the growth medium with a myogenic differentiation medium (DMEM with 2% EV-depleted horse serum (Thermofisher Scientific, Altrincham, UK)). The cells were maintained at 37 °C in 5% CO_2_. Differentiation medium was changed every other day. The time points used in experiments were 24 h, 2 days, 3 days and 4 days. Cell morphology was monitored by phase contrast microscopy. After differentiation, C2C12 cells could be maintained in low serum medium (DMEM with 2% EV-depleted FBS) without de-differentiation and this was used in co-culture experiments with CMT 64/61 cells. To deplete EVs from the FBS, 2 or 5% FBS was diluted 1:1 with PBS and centrifuged at 100,000× *g* for 18 h, collecting the supernatant.

### 2.2. Extracellular Vesicle (EV) Isolation from Cell Culture (Conditioned) Medium and EV Characterisation

C2C12 myocytes and NIH-3T3 fibroblast cells were grown in complete growth medium (CGM) until 70% confluent and then washed three times with pre-warmed PBS and incubated with 10 mL serum-free medium (SFM) for 16 h at 37 °C in 5% CO_2_ to avoid contamination with FBS-derived EVs for analysis of the cell-derived EVs. After 16 h, the SFM was collected from the cell cultures (98% viable by ViaCount assay; see Section 2.9.1) for EV analysis: first it was centrifuged at 300× *g* for 15 min to remove dead cells and debris, then 18,500× *g* for 1 h at 4 °C to remove apoptotic bodies. The resultant supernatant was centrifuged at 85,000× *g* for 90 min at 4 °C. The isolated EV pellet was resuspended in filtered (0.2 μm pore size) PBS and centrifuged again at 85,000× *g* for 90 min at 4 °C to remove proteins and other low molecular weight factors bound to the EV membrane surface. The final EV pellet was then resuspended in cold filtered (0.2 μm) DMEM. The resulting EV population therefore consisted of small-sized EVs (sEVs) which were used in subsequent experiments. The protein concentration of the EVs was determined using BCA (Sigma-Aldrich) and NanoOrange (Thermo Scientific) protein assay kits and used to calculate the amount of EVs used in all experiments. Further characterisation of EVs by NTA, surface marker detection, dynamic light scattering (DLS) (to ascertain polydispersity and zeta potential), and transmission electron microscopy was carried out in accordance with MISEV2018 and 2023 guidelines [46,47].

The concentration of EVs per sample and size distribution profiles of EVs isolated were analysed using the NanoSight NS300 system (Malvern Instruments Ltd., Malvern, UK) configured with a sCMOS camera and a Blue488 laser. For NTA analysis, EVs collected from 3.2 × 10^7^ C2C12 myocyte and NIH-3T3 fibroblast cells were used, and five different samples at different passages were collected. EVs were diluted 1/500 in DPBS to a final volume of 1 mL before application on the NanoSight. Following the manufacturer’s software manual (Malvern Instruments Ltd.), five 1 min videos were captured at a temperature range of 17.8–19.2 °C. The videos were analysed by the in-built NanoSight Software NTA 3.2 (Malvern) to assess EV concentration and size-distribution profiles. Where depletion of EVs from CM was required, CM was centrifuged for 18 h at 120,000× *g* for 18 h and then ultrafiltration performed (amicon 100 kDa; 1 h/3500× *g*). The % depletion of EVs was calculated by NTA. For DLS, EVs were diluted in PBS and measurements were performed using a Malvern Zetasizer Nano ZS (Malvern Instruments Ltd.) to determine size, polydispersity index (PDI) and zeta potential. At least ten spectra were made from three replicates, at 22 °C, and averages calculated.

C2C12 myocyte EVs were imaged by TEM, following negative staining according to previously described methods, using aqueous uranyl acetate (2%) with aqueous bacitracin [48]. The EV samples were carefully placed on 400-mesh copper grids coated with thin Pioloform support film (Agar Scientific Rotherham, UK) and then pre-treated with aqueous Alcian Blue 8GX (Thermo Fisher Scientific). Digital images were captured on an AMT XR16 CCD camera using the JEOL JEM 1200 EX II transmission electron microscope. (JEOLLtd., Akishima, Tokyo, Japan).

### 2.3. Light Microscopy and Cell Counting

Cells were observed by light microscopy (Nikon Eclipse Inverted Microscope, TS100, Nikon, Tokyo, Japan) using the 10× objective to assess and ascertain morphological changes from the undifferentiated myoblast state to the differentiated myocyte state. Cell count and concentration were determined using a haemocytometer, following staining with 0.4% trypan blue (Thermofisher Scientific). Cell counts were performed in triplicate and analysed under a 10× objective, calculating the number of viable cells per ml.

### 2.4. Fluorescent Microscopy

Microscopic observation of cells was typically carried out with CMT 64/61 cells cultured in 6-well microscopic chambers that had been treated with C2C12-EVs, washed with PBS, incubated with the specific fluorescent dyes, washed (×3) and then fixed in 4% paraformaldehyde (37 °C for 15 min). The cell culture chamber was separated from the microscope slide, air dried and the coverslips mounted on the microscope slide using DAPI-VECTASHIELD medium (ThermoFisher Scientific). Images were then collected using an inverted fluorescent microscope (EVOS™ FL Digital Inverted Fluorescence Microscope, Invitrogen™, Carlsbad, CA, USA).

### 2.5. PKH67 Labelling of CMT 64/61 Murine Lung Carcinoma Cells

PKH67 labelling was carried out according to the manufacturer’s instructions (Sigma-Aldrich). Once at 90% confluency, CMT 64/61 cells (2 × 10^7^) were washed in SFM and centrifuged at 400× *g* for 5 min. After centrifugation, the cell pellet was resuspended in 1 mL of diluent C to prepare a 2× cell suspension. PKH67 dye (4 μL) was mixed with 1 mL of diluent C and added to the cell suspension, the mixture being incubated for 5 min with mixing. Staining was stopped by adding 2 mL of 1% BSA (Sigma-Aldrich) with incubation for a further 1 min. The cell suspension was then washed (400× *g* for 10 min/25 °C), resuspended in 10 mL CGM and re-centrifuged; this procedure was repeated three times to remove unincorporated dye. After cell viability was assessed, 1 × 10^4^ cells were cultured in glass bottom optical plates (ThermoFisher Scientific). The plates were incubated at 37 °C/5% CO_2_ for 24 h and visualised using the EVOS™ Digital Inverted Fluorescence Microscope.

### 2.6. PKH26 Labelling of EVs for EV Tracking

EVs isolated from the C2C12 myocytes were labelled with PKH26 dye according to the manufacturer’s instructions (Sigma-Aldrich). EVs (25 μL of 100 μg/mL) resuspended in PBS were mixed with 250 µL of diluent C. PKH26 dye (2 μL) was then mixed with 250 µL of diluent C and added to the EV suspension, mixed and left for 5 min on ice. Staining was stopped with 500 µL of 1% BSA and placed on ice for 1 min. To remove unincorporated dye, size exclusion chromatography was performed by running the PKH26-EVs through a Sepharose CL-2B mini-column (PD10, Cytiva; bed volume: 8.3 mL, diameter 1.5 cm) with a bottom frit to retain the Sepharose and top frit to stop the column from drying, using Dulbecco’s Phosphate-Buffered Saline (DPBS) as eluent. After the sample was loaded, 1 mL eluted fractions were collected and measured for fluorescence using a spectrofluorometer. The fractions containing labelled EVs were typically the fourth or fifth fraction and concentrated by ultrafiltration (100 kDa MW cut-off). To make a temporal quantification of PKH-labelled C2C12-EVs’ uptake by CMT 64/61 cells, the EVs were incubated with CMT 64/61 cells for 1 h. After an acid wash in glycine buffer to remove surface EVs not internalised, the percentage of cells with internalised EVs was determined by flow cytometry and the kinetics of uptake monitored over 12 h.

### 2.7. Interaction Analysis of Labelled C2C12-EVs with CMT 64/61 Murine Lung Carcinoma Cells

Murine lung carcinoma cells (CMT 64/61) were labelled with PKH67 (fluorescent green) and 1 × 10^4^ cells/well were grown for 16 h on glass coverslips placed into a 12-well plate. The CMT 64/61 cells were then treated with 200 ng/mL of PKH26- (fluorescent red) labelled C2C12 myocyte EVs (“EV transplant”). Carcinoma cell nuclei were counterstained with Hoechst-33342 stain and the cells were analysed by fluorescent microscope (EVOS™) at different time intervals (2, 6 and 12 h, respectively) to assess EV uptake into the cells.

### 2.8. Cell Growth Assay and Assessment of Conditioned Medium or EV Transplant on CMT 64/61 Viability

To assess the effects of C2C12 myocyte-conditioned medium (CM) or EVs on murine lung carcinoma cells (CMT 64/61), the cells were seeded in 24-well plates (at 2 × 10^4^ cells/mL/well) in triplicate and incubated for 24 h at 37 °C. Cell attachment to the plates was observed and confirmed under a light microscope and the cells then washed three times with pre-warmed PBS. Each well of CMT 64/61 cells was treated with the appropriate CM, concentrated from 15 mL to 300 μL by ultrafiltration (100 kDa cutoff), as well as with EVs (“EV-transplant”) at different protein concentrations (100, 200, 300 and 500 ng/mL of EVs, respectively). The volume of SFM was adjusted to 500 µL per well and the plates were thereafter incubated at 37 °C for 48 h. Triplicate wells with untreated cells in 5% EV-depleted FBS was used as a control. Other controls included untreated negative control; soluble factors (CM without cells/EVs) removed by size exclusion chromatography and ultrafiltration (SEC-UF). The supernatant from each well was then transferred to 1.5 mL micro-centrifuge tubes (one tube for each triplicate treatment). The wells were washed twice with DPBS and detached with trypsin (25 μL of 0.25% Trypsin/EDTA) at 37 °C for 5 min. The cells were resuspended in 175 μL of CGM, collected into micro-centrifuge tubes and the cell viability determined by flow cytometry using the Guava ViaCount Assay (Luminex corporation).

### 2.9. Flow Cytometry

The Guava flow cytometer (Guava^®^ EasyCyte 8HT, Millipore, Bracknell, UK was used to perform three different assays: the ViaCount assay (for counting cells and determining viability), the Nexin assay (for reporting apoptosis) and the cell cycle analysis assay (for assessing cell cycle arrest).

#### 2.9.1. ViaCount Assay—Cell Viability Assessment

Cell number and viability were determined using the Guava ViaCount assay (Cytek Biosciences Ltd., Ely, UK) which distinguishes viable from non-viable cells according to the differing permeabilities of two DNA-binding dyes. Cells (1 × 10^4^ per well) were seeded in triplicate into a 24-well plate, with or without C2C12-EVs, and treated with 5% EV-depleted FBS and incubated for 48 h. The cells were detached using trypsin, washed, collected and replicates combined to achieve the desired cell concentration per test. Stained cell samples were prepared by mixing 20 µL of cell suspension with 180 µL of Guava ViaCount reagent in the wells of a 96-well plate. The cell viability assay was performed using a Guava EasyCyte 8HT flow cytometer. For the zero-EV control, average and SD was calculated. The data was then normalised according to the control average.

#### 2.9.2. Nexin Assay—Apoptosis Assessment

The CMT 64/61 carcinoma cells, treated with or without myocyte EVs, as described above for the cell viability protocol, were detached with trypsin and washed. The cells were then treated with 1% FBS and 100 µL of Nexin reagent comprising (phosphatidyl-serine- (PS-) binding Annexin V-phycoerythrin and the cell-permeant fluorescent DNA intercalator dye, 7-AAD (Cytek Biosciences Ltd.). The plate was incubated for 20 min at RT and analysed using the Guava EasyCyte 8HT flow cytometer (Millipore).

#### 2.9.3. Cell Cycle Assay—Assessment of Cell Cycle Arrest

To assess the different cell cycle phases of the carcinoma cells treated with EVs, a Guava cell cycle assay was used. Treated cells were incubated with 5% EV-depleted FBS for 24 h. On the day of the experiment, the cells were detached, washed and transferred to a 96-well plate where the cell pellet was resuspended in 200 μL ice-cold 70% ethanol. The plate was sealed and refrigerated for 12 h, washed in PBS (300 g/5 min) and incubated with 200 μL Guava cell cycle reagent (propidium iodide) for 30 min/RT in the dark. The samples were then analysed on the Guava EasyCyte 8HT flow cytometer using the ModFit software, version 5.0 (https://www.vsh.com/products/mflt/, accessed on 19 March 2021).

### 2.10. Cell Migration Assay

The murine lung carcinoma cells (CMT 64/61) were seeded in triplicate at 1 × 10^5^ cells in Corning 8.0 μm Transwell^®^ Inserts (Thermofisher Scientific) with SFM (DMEM). C2C12-EVs were added (100 and 200 ng/mL; “EV-transplant”) to the recipient wells in the transwell 12-well plates with 5% FBS as a chemoattractant. EV-free recipient wells were used in triplicate as background control. After incubation at 37 °C for 24 h, the medium from both recipient wells and the inserts were aspirated. The inserts were washed twice with PBS and cells that had adhered to the inside of the insert were removed using a cotton swab and the insert washed again with PBS. Crystal violet stain (Thermofisher Scientific) was used to stain the cells adhered to the insert membrane and, for quantification, the cells from 5 random areas were counted under the light microscope. To ascertain the number of CMT 64/61 cells that had migrated per field of view (FOV), cells were counted from 7 image fields, using the ImageJ 1.x (Fiji)multi-point tool (https://imagej.net/ij/, accessed on 19 March 2021).

### 2.11. Cell Cytotoxicity Assay

CMT 64/61 cells were seeded in triplicate in 96-well plates (at 1 × 10^4^ cells/well) and treated with 5% EV-depleted FBS-containing growth medium with the different EV concentrations as before (at 37 °C for 24 h). Triplicate wells without cells served as a negative control to determine the culture medium background. A second set of triplicate wells with untreated cells served as an additional control and a third set of triplicate wells with untreated cells was prepared to enable determination of the maximum lactate dehydrogenase (LDH) release (positive control). Aliquots (50 µL) from all test and control wells were transferred to a fresh 96-well glass bottom optical plate and 50 µL of CytoTox 96^®^ reagent (Promega, Chillworth, UK) added to each aliquot. The plate was covered with foil and incubated for 30 min at RT. To generate a maximum LDH release, 10 µL of 10 × lysis solution was added to the positive control wells 45 min before adding the CytoTox 96^®^ reagent. Finally, 50 µL of stop solution was added to each sample and absorbance recorded at 492 nm on a FLUOstar Omega microplate reader (BMG Labtech, Ortenberg, Germany). The percentage of cytotoxicity was calculated using the following formula:

$$Percent\;Cytotoxicity=\frac{Experimental\;LDH\;release}{Maximum\;LDH\;release}\;\times 100$$

### 2.12. LysoTracker™ Analysis

The status of cell lysosomes in the CMT 64/61 cells treated with the C2C12 “EV transplant,” was assessed using LysoTracker™ analysis (Thermofisher Scientific). The CMT 64/61 cells (1 × 10^4^ cells/well), which had been treated with 5% EV-depleted FBS with or without C2C12-EVs, was assessed for the status of lysosomes with 5000 cells from each well transferred to a 96-well plate. After incubation with 75 nM LysoTracker™ (37 °C/45 min), the cells were washed, and lysosomal fluorescent intensity was measured on a FLUOstar Omega microplate reader (Excitation/Emission: 504 nm/511 nm).

### 2.13. MitoTracker™ Analysis

MitoTrackerTM was used to assess the status of mitochondria in CMT 64/61 cells treated with, or without, C2C12-EVs, as before (“EV-transplant”). In total, 5000 cells from each well in 96-well plates were resuspended for 30 min in pre-warmed growth medium containing 100 nM MitoTracker™ (Thermofisher Scientific). The cells were washed, and fluorescent intensity then measured using the FLUOstar Omega microplate reader (Excitation/Emission: 579/599 nm). For fluorescent microscopy, cells were stained, then incubated in 4% *v*/*v* formaldehyde in PBS for 15 min and washed in PBS before visualisation.

### 2.14. Caspase 3, Caspase 8 and Caspase 9 Multiplex Activity Assay

After treating CMT 64/61 cells with C2C12-EVs for 24 h at 37 °C, stimulation of apoptosis was determined using the fluorescent caspase 3, caspase 8 and caspase 9 Multiplex Activity Assay Kit (Abcam, Cambridge, UK). The loading solution (100 μL) was added to each well of a sterile black 96-well plate with a clear flat bottom and incubated at RT in the dark for 1 h. As a positive control, cells were treated for 48 h at 37 °C with 100 μM H_2_O_2_. After incubation, fluorescent intensity was measured using the FLUOstar Omega microplate reader (BMG Labtech) in bottom read mode (caspase 3: Ex/Em = 535/620 nm (red), caspase 8: Ex/Em = 490/525 nm (green), caspase 9: Ex/Em = 370/450 nm (blue)).

### 2.15. SDS-Polyacrylamide Gel Electrophoresis and Western Blotting

Proteins were extracted from C2C12 myoblast cell lysates (after 24 h incubation) and from C2C12 cells in differentiated media (24 h and 4 days incubation), respectively. In brief, the detached cells were lysed with ice-cold RIPA buffer (Sigma-Aldrich) containing HaltTM protease inhibitor cocktail (Thermofisher Scientific) for 2 h on ice (1 mL/10^7^ cells), according to previously published methods [49]. Thereafter, proteins were collected by centrifugation at 16,000× *g* for 20 min at 4 °C. The same method was used where needed to extract proteins from the EV preparations. Protein concentrations of cell lysates or of EVs were determined using the BCA Protein Assay Kit (Thermo Scientific) or NanoOrange (Thermo Scientific), measuring absorbance at 562 nm using a FLUOstar Omega microplate reader (BMG Labtech). SDS-PAGE and Western blotting was carried out using the Mini-PROTEAN tetra vertical electrophoresis system (Bio-Rad, Watford, UK) and 4–20% TGX gels (Bio-Rad). Samples were reconstituted 1:1 in 2 × Laemmli sample buffer containing 5% β-mercaptoethanol (Bio-Rad, UK), boiled at 100 °C for 5 min and separated by SDS-PAGE at 165 V. For semi-dry Western blotting, proteins were transferred onto 0.45 μm nitrocellulose membranes (Bio-Rad) for 1 h at 15 V and even protein transfer was assessed using Ponceau S staining (Sigma, Gillingham, UK). Membranes were blocked with 2% semi-skimmed milk or 5% BSA (BSA, Sigma-Aldrich) in Tris-buffered saline containing 1% *v*/*v* Tween20 (TBS-T) for 1 h at RT. Primary antibody incubation was carried out on a shaking platform at 4 °C overnight. The following primary antibodies were used: anti-myogenin (F5D, 1/1000, Abcam); anti-CD63 (1/1000, ab216130, Abcam); anti-TSG101 (4A10, 1/1000 Novus Biologicals, Bio-Techne Ltd, Abingdon); anti-Decorin (AF1060, 1/1000; R&D systems, Bio-Techne); GM130 (1/2500 recombinant Rabbit MAb (2L3P6); ThermoFisher Scientific); anti-β-actin (ab8226, 1 μg/mL, Abcam); anti-Bcl-2 (clone 100/D5, 1/200, Thermo Fisher); anti-Bax (clone 6A7; 1/500; Sigma-Aldrich). The membranes were washed in TBS-T for 3 × 10 min, incubated with secondary IgG HRP-conjugated antibodies (Bio-Rad, 1:3000 dilution; 1 h/RT), washed five times in TBS-T and then visualised using a UVP BioDoc-ITTM System (Cambridge, UK) in conjunction with ECL (Cytivia, Amersham, UK).

### 2.16. LC-MS/MS Analysis

The protein concentration of EVs from C2C12 myocytes and NIH-3T3 fibroblasts was determined as described above. The protein samples were run 0.5 cm into a 12% TGX gel (BioRad) and the concentrated band of proteins was excised for in-gel digestion and LC-MS/MS analysis (Cambridge Centre for Proteomics, Cambridge, U.K.). The protein samples were digested with trypsin and analysed using the Dionex Ultimate 3000 RSLC nanoUPLC and Q Exactive Orbitrap mass spectrometer system (Thermo Fisher Scientific Inc, Waltham, MA, USA). Reverse-phase chromatography was carried out using a Thermo Scientific reverse phase nano Easy-Spray column (Thermo Scientific PepMap C18, 2 μm particle size, 100 Å pore size, 75 μm i.d. × 50 cm length) to separate peptides. The isolated peptides were loaded onto a pre-column (Thermo Scientific PepMap 100 C18, 5 μm particle size, 100 Å pore size, 300 μm i.d. × 5 mm length) from the Ultimate 3000 autosampler with 0.1% formic acid for 3 min at a flow rate of 10 μL/min. The elution of peptides from the pre-column was then switched onto an analytical column and facilitated by switching the column valve (solvent A = water + 0.1% formic acid; solvent B = 80% acetonitrile, 20% water + 0.1% formic acid). A linear gradient of 2–40% of solvent B was employed for 30 min. The LC eluate was sprayed into the mass spectrometer using the Easy-Spray source (Thermo Fisher Scientific Inc.). All m/z values of eluting ions were measured in an Orbitrap mass analyser, set at a resolution of 70,000 and scanned between m/z 380–1500. Data-dependent scans were employed to automatically isolate and generate fragment ions by higher energy collisional dissociation (HCD, NCE: 25%) in the HCD collision cell. The resulting fragment ions measurements were performed using the Orbitrap analyser, set at a resolution of 17,500. Ions that were singly charged and ions with unassigned charge states were excluded from being selected for MS/MS, and a dynamic exclusion window of 20 s was employed. The MS/MS data was converted to mgf files and analysed using Mascot (Matrix Science, London, UK; version 2.6.0), searching against the UniProt *Mus musculus* database (55,474 protein sequences). Ions score for hit identification was set at −10*Log(P), where P was the probability that the observed match was a random event, with individual ions scores >35 indicating identity or extensive homology (*p* < 0.05). The fragment and peptide mass tolerances were set to 0.1 Da and 20 ppm, respectively.

### 2.17. Protein Identification, Protein–Protein Interaction Networks and Pathway Enrichment Analysis

Scaffold (version Scaffold_4.11.1, Proteome Software Inc., Portland, OR, USA) was used to validate MS/MS-based peptide and protein identifications. Peptide identifications were accepted if they could be established at a >95.0% probability by the Peptide Prophet algorithm with Scaffold delta-mass correction and contained at least 5 identified peptides. The significance threshold was set at *p* < 0.05. Proteins containing similar peptides that could not be differentiated according to MS/MS analysis alone were grouped. Proteins were annotated with GO terms from NCBI (downloaded 14 January 2023). Pathway annotations were fetched from https://reactome.org/. STRING analysis (https://string-db.org/; accessed 15 September 2024) was furthermore used to generate protein–protein interaction networks based on the protein IDs of the C2C12 myotube and NIH 3T3 EVs, respectively. The “*Mus musculus*” species database was used in STRING, with the minimum required interaction score set to “medium confidence” (0.400) and KEGG (Kyoto Encyclopaedia of Genes and Genomes) pathway enrichment analysis was carried out.

### 2.18. Statistical Analysis

Statistical analysis for all data was performed using the unpaired *t*-test for repeated measures or 1-/2- way ANOVA using GraphPad Prism version 6.01 for Windows (GraphPad Software, San Diego, CA, USA). Throughout, experiments represent 3 biological replicates (*n* = 3) and 3 technical replicates (*N* = 3) and the mean ± SD is reported. Statistical correlations between data values were also determined using GraphPad Prism software. Differences giving a value of *p* < 0.05 with confidence interval of 95% were considered statistically significant.

## 3. Results

### 3.1. Myoblast C2C12 Cell Differentiation and Extracellular Vesicle (mEV) Isolation from Myocytes

The murine myoblast cell line, C2C12, was differentiated to myocytes at low serum concentrations. Myogenic differentiation was induced by switching the cells to skeletal muscle differentiation medium containing 2% EV-depleted horse serum. After 2 days, the myoblast cells (mono-nucleated and with a flattened, star-shaped morphology) (Figure S1A) were beginning to change shape and their typical monolayer organisation. By day 3, they were elongated and packed closely together and by day 4, in the intermediate stage of myogenesis, cells were beginning to fuse to form nascent myotubes (Figure S1B). Confirming differentiation, the cells started to express the myocyte-specific protein, myogenin (Figure S1C) after 24 h and fully by day 4, when myotubes were formed, in 2% horse serum differentiation medium (DM), but not in medium supplemented with FBS (GM).

Assessment of EV profiles by NTA confirmed a heterogenous C2C12-EV population with three main peaks at 137.7 ± 5.60 nm, 228 ± 17.04 nm and 337.67 ± 25.52 nm (Figure S2A). As the sEVs were isolated from conditioned medium without FBS, contamination with bovine EVs was unlikely. The peak modal size, 137 nm, was close to the DLS average size, 121 nm (Figure S2C). The low PDI value at 0.2 suggested the isolation was largely effective at removing contaminants such as protein aggregates or other debris. The zeta potential for the C2C12-EVs (Figure S2D) was determined as −19 mV which indicated moderate stability and repulsive forces that might limit aggregation. The EVs were further visualised with transmission electron microscopy (TEM) (Figure S2E). Based on the NTA analysis, and confirmed by TEM, the mean size of EVs was 174.7 ± 2.9 (Figure S2B). The D10, D50 and D90 reported in Figure S2B indicate the sizes below which 10%, 50% and 90% of the EVs fall. This confirmed that the EVs isolated were small EVs (sEVs). The yield of these EVs isolated by dUC was 8.81 × 10^10^ ± 2.74 ×10^8^ particles/mL. The calculated number of EVs/mL: μg/mL protein ratio gave a value of 2.9 × 10^10^ EVs/μg, indicating a pure EV population (low non-EV protein contamination). Expression of the EV markers CD63 (transmembrane protein) and TSG101 (cytosolic protein) was also confirmed (Figure S2F), but not of the EV contaminant protein, GM130. In experiments where EVs and soluble factors in CM from C2C12 culture needed to be isolated separately, size exclusion chromatography was carried out as described above (Section 2.6). The EVs (CD81^+^ by western analysis) were effectively isolated from soluble proteins, and mainly found in fractions 6–10. The main fractions containing protein (Soluble Factors) were 12–14 but also co-eluted EVs, likely as certain EVs may be small enough to be of similar size to protein aggregates (Figure S2G).

### 3.2. The Murine Lung Carcinoma (CMT 64/61) Cell Line Grows Rapidly in Low Serum Conditions

The highly metastatic murine lung carcinoma cell line CMT 64/61 maintained a similar, but reducing, growth rate when grown in Waymouth’s MB 752/1 medium (WM) in 10%, 5% and down to 2% serum (FBS) for the first 48 h (Figure S3A). Thereafter, as expected, up to day four there were increasing differences in growth when comparing cells grown in 10% FBS with those grown in low serum conditions. Of relevance to co-culture and transwell experiments in which CMT 64/61 (adapted to DMEM) were maintained in DMEM and 10% FBS, (Figure S3A), 2% FBS (even EV-depleted) only marginally reduced growth over 48 h (*p* = 0.053). Compared to 5% FBS, in 5% EV-depleted FBS (diluted 1:1 with PBS and centrifuged at 100,000× *g* for 18 h), the cell numbers were not significantly lower by 48 h (*p* = 0.143). We also found that proliferation of CMT 64/61 cells was non-significantly lower at 48, 72 and 96 h growth in DMEM than WM (both with 2% EV-depleted FBS).

### 3.3. Lung Carcinoma (CMT 64/61) Cells Fail to Colonise Skeletal Muscle Cells, and CMT 64/61 Viability Is Decreased Following Treatment with Myocyte C2C12-Derived EVs (“EV Transplant”)

In preliminary experiments using an in vitro cell culture approach, the fate of murine lung carcinoma cells (CMT 64/61) introduced to differentiated C2C12 skeletal muscle cells (myocytes), at a ratio of 0.025:1 or 0.25:1, was investigated (Figure S4). The C2C12 myocytes were grown to confluency, then washed and co-cultured with the CMT 64/61 carcinoma cells in DMEM with 2% EV-depleted FBS. The murine embryonic fibroblast cell line (NIH-3T3), known to facilitate and enhance cancer cell proliferation [50] and invasion [51], was used as a control cell line for the myocytes. Use of EVs from this non-muscle cell type enabled us to distinguish those effects specific to C2C12 myocytes from more general effects. Even though C2C12 myocytes are known to support the growth of certain cell types, including neuronal PC12 and 3T3-L1 adipocytes (although not confirmed here), after 48 h, the CMT 64/61 lung carcinoma cells failed to colonise the C2C12 cells at both ratios tested (CMT 64/61:C2C12; 0.025:1 and 0.25:1), and the morphology of the myocytes remained unchanged (Figure S4B,C). As the CMT 64/61 cells were in DMEM rather than the optimal Waymouth’s MB 752/1 (WM), we ascertained that growth of CMT 64/61 was not compromised by the change in medium (filled versus unfilled green boxes in Figure S3A). In the controls, where the CMT 64/61 carcinoma cells were co-cultured with NIH-3T3 fibroblasts, the cancer cells significantly invaded and colonised the fibroblast cells, even at the lower ratio tested (CMT 64/61:NIH-3T3 of 0.025:1). The normal morphology of NIH-3T3 fibroblasts was visibly affected by the carcinoma cells, showing cellular dysplasia (Figure S4E,F). Furthermore, a small area of new cell growth was seen, within the yellow dotted line shown in Figure S4F for CMT 64/61: NIH-3T3 (0.25:1) and comparison of these cells’ morphology with those in Figure S4G (CMT 64/61 cells grown under normal conditions) suggested carcinoma cell invasion of the fibroblasts within 48 h. Conversely, the results suggested that myocytes inhibited such invasion/colonisation by the CMT 64/61 cells.

As previous studies had indicated that host microenvironments can promote cancer metastasis and progression [52,53], in crude experiments we first assessed whether the myocyte-conditioned medium (CM) could exert inhibitory effects on CMT 64/61. We found that after 48 h incubation with CM from the C2C12 cells, the viability of CMT 64/61 cells was significantly decreased, in a dose-dependent manner (Figure S4H, red bars), whereas CM from NIH-3T3 fibroblasts slightly increased their viability (Figure S4H, green bars). Furthermore, in the presence of increasing concentrations of C2C12 CM, the morphology of the CMT 64/61 cells changed (Figure S4I), displaying fewer cell colonies with fewer cells (white arrows). At a concentration of 5 μg/mL of C2C12 CM, there was a loss of CMT 64/61 cell membrane integrity and of cell shape (yellow arrows), as well as indication of membrane blebbing. In comparison, in the presence of control NIH-3T3 CM the carcinoma cells increased in number and decreased in size (Figure S4I) as they overgrew in multilayers (3 and 5 μg/mL NIH-3T3 CM).

**Figure 1:**
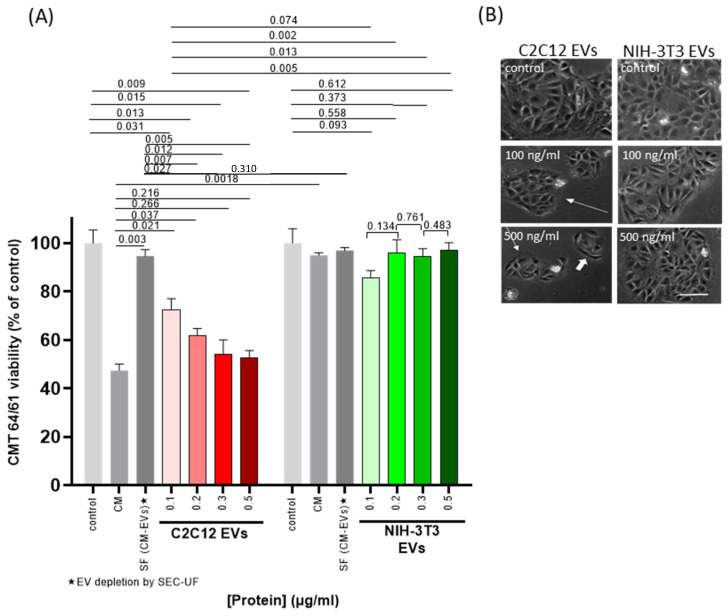
EVs from C2C12 myocyte cells inhibit proliferation of CMT 64/61 lung carcinoma cells. Cell viability analysis of CMT 64/61 carcinoma cells treated with C2C12 and NIH-3T3 EVs. (**A**). EVs at a range of concentrations were added to CMT 64/61 cells (48 h/37 °C), cells were stained with Guava ViaCount reagent and analysed by flow cytometry. DC, differential centrifugation; SEC-UF, size exclusion chromatography–ultrafiltration. Control = zero-EV control; CM = conditioned medium (EVs + soluble factors (SF)); SF = soluble factors. The data presents the mean ± standard deviation of three independent experiments performed in triplicate; *p* < 0.05 was considered statistically significant. Representative photomicrographs from two of the three independent experiments of CMT 64/61 cells treated with increasing protein concentrations of C2C12 and NIH-3T3 EVs (48 h; 37 °C). (**B**). scale bar = 100 μm. Whereas, after 48 h in the presence of NIH-3T3 EVs, the number of cell colonies and the cells within these colonies increased, with C2C12-EVs, some of the CMT 64/61 cells appeared elongated (white arrows), rather than the typical polygonal shape and presented fewer cell clusters with decreased numbers of cells.

The same set of experiments was repeated now using EVs isolated from the C2C12 CM (“EV-transplant”). After co-culturing for 48 h, cell viability was assessed using Guava ViaCount as before. Incubation with the C2C12-EV isolates showed a dose-dependent decrease in CMT 64/61 cell viability (even at 100 ng/mL) (Figure 1A, red bars), but not when applying EVs from the NIH-3T3 CM (Figure 1A, green bars). To control for the effect of soluble factors (SFs) in the CM, EVs were depleted from CM by SEC and ultrafiltration (SEC-UF). The % reduction was calculated by NTA to be 96% (8.8 × 10^10^ EVs/mL to 3.5 × 10^9^ EVs/mL). EV-depleted SFs from C2C12 culture only marginally reduced the numbers of viable CMT 64/61 compared to control (dark grey bars in Figure 1A, *p* = 0.242) and C2C12-CM significantly reduced CMT 64/61 viability compared to C2C12-SF (*p* = 0.003). Compared to C2C12-CM, C2C12-EVs at 300 and 500 ng/mL inhibited CMT 64/61 viability to a similar, non-significantly different level. Compared to the lower 100 and 200 ng/mL concentrations of C2C12-EVs, however, C2C12-CM reduced viability to a further extent. At all concentrations of C2C12-EVs, CMT-64/61 viability was significantly reduced compared to C2C12-SF (CM depleted of C2C12-EVs) and to all NIH-3T3-EV concentrations. It was also noted that C2C12-CM reduced CMT 64/61 viability to a significantly lower level than NIH-3T3-CM (*p* = 0.002), but not comparing C2C12-SF with NIH-3T3-SF (*p* = 0.310). Microscopy of CMT 64/61 cells exposed to myocyte EVs showed fewer cell colonies (long arrows) and cells showing shrinkage, cytoplasmic blebbing and poor attachment (Figure 1B). The number of cells within the colonies and their size was also reduced (short white arrows) compared to untreated controls. As a control to show the specificity of the anti-cancer effect of C2C12-EVs, the carcinoma cells were also treated with fibroblast (NIH-3T3) EVs. The treated CMT 64/61 cells which had shown no significant change in viability (Figure 1A) also showed no overt change in morphology or signs of apoptosis (Figure 1B).

Our results indicated that C2C12-EVs reduced the viability of CMT 64/61 cells, in accordance with previous reports of skeletal muscle-derived media and their effects on carcinoma cells [54,55]. The data also suggests that the negative effect of myocyte EVs on carcinoma cell viability is similar to that mediated by a 10-fold greater protein concentration of myocyte CM.

### 3.4. Anti-Cancer Effects of Skeletal Muscle-Derived EVs on Lung Carcinoma (CMT 64/61) Cells

#### 3.4.1. C2C12-EVs Inhibit Lung Carcinoma (CMT 64/61) Cell Migration

**Figure 2:**
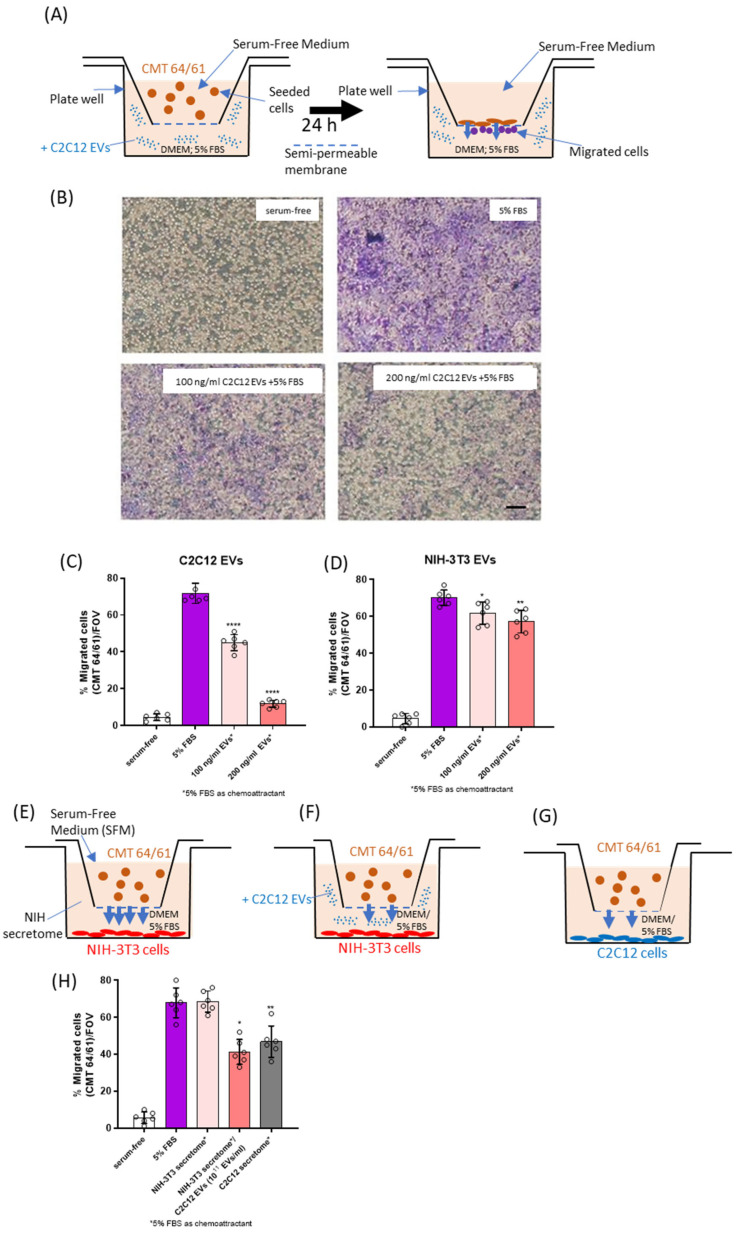
C2C12-EVs reduce lung carcinoma CMT 64/61 cell migration. (**A**) A schematic diagram of the CMT 64/61 cell migration model in which CMT 64/61 lung cancer cells were plated in the upper chamber and during the incubation, cells move to the lower compartment in response to signals from the chemoattractant. (**B**) Representative micrographs of carcinoma cell transwell migration. Serum-free media and 5% FBS-containing medium were added to the lower chamber as the negative control and chemoattractant (positive control), respectively. Different concentrations of myocyte C2C12-EVs with 5% FBS were used as test samples. After cell migration and staining with crystal violet, the migrated cells (purple stained) were counted as described in Materials and Methods. Scale bar = 100 µm. The membrane pores were observed as numerous small round white coloured dots in the micrograph (especially visible in panel B (serum-free)). (**C**,**D**), migrated cells were counted. In (**E**,**H**) migration of CMT 64/61 cells to the lower chamber containing the NIH-3T3 secretome was monitored. In (**F**,**H**) similar migration to the NIH-3T3 secretome supplemented with C2C12-EVs was monitored and in (**G**,**H**) migration was to the C2C12 secretome. Data represent the mean ± SD from three separate experiments. Unpaired t- test was conducted with EVs treated and 5% FBS-treated samples (positive control). *p* ≤ 0.05 was considered to be significant and noted between positive control and EV-treated samples (* *p* ≤ 0.05; ** *p* ≤ 0.01; **** *p* ≤ 0.0001).

To assess the effect of C2C12-EVs on the migration of lung carcinoma (CMT 64/61) cells, a transwell cell migration assay was performed (Figure 2A). In the presence of the chemoattractant (5% FBS), CMT 64/61 cell migration was significantly increased after 24 h, compared to serum-free medium (SFM) (Figure 2B–D). However, when applying EVs derived from the C2C12 myocytes (at 100 and 200 ng/mL) a dose-dependent decrease in CMT 64/61 cell migration towards the chemoattractant was observed (Figure 2B,C). Incubation with 100 ng/mL C2C12-EVs promoted a 1.4-fold decrease in cell migration, and 200 ng/mL promoted a 2.6-fold decrease in cell migration. In comparison, incubation with EVs derived from the control fibroblast NIH-3T3 cells showed significantly greater migration than in the case of the C2C12-EV transplant (Figure 2D). When CMT 64/61 cells in the upper chamber were exposed to the NIH-3T3 secretome in the lower chamber, CMT 64/61 cells migrated into the lower chamber (Figure 2E,H). However, when the NIH-3T3 secretome in the lower chamber was supplemented with 10^11^ C2C12-EVs/mL (100 ng/mL), there was reduced CMT 64/61 migration (Figure 2F,H). CMT 64/61 cells were inhibited from migrating to the C2C12 secretome (Figure 2G,H) to a similar extent, even though the concentration of C2C12-EVs measured by NTA was 10-fold lower than after supplementation (10^10^ EVs/mL). That the C2C12 secretome-including EVs inhibited CMT 64/61 migration to a similar level as C2C12-EVs alone further supports that the EVs have the prominent role but also suggests that other components of the secretome may also affect CMT 64/61 migration.

We considered the FBS-induced migration of CMT 64/61 cells to be due to effects on its migration, rather than any effect FBS may have in increasing cell growth, as the proliferation of these cells in 5% FBS was not significantly affected compared with 5% EV-depleted FBS, over a period of two days (Figure S3). The results from these experiments point to possible anti-cancer roles of skeletal muscle (myocyte) EVs (C2C12-EVs) that may partly explain the rarity of cancer metastasis to skeletal muscles.

#### 3.4.2. C2C12-EVs Are Taken up by Lung Carcinoma (CMT 64/61) Cells

Research from our own and other groups has shown EV interaction, for example, by membrane fusion, between various cell types, including microbial and immune cells. However, EVs may also be taken up into recipient cells where they release their intra-vesicular content and induce molecular changes [49,56]. As our findings above showed myocyte-derived EVs to reduce carcinoma cell viability and migration, we wanted to address the nature of the interaction between these myocyte EVs and the cancer cells. For this purpose, we utilised fluorescent dyes commonly used for in vitro and in vivo cell tracking, which do not alter cell functions such as proliferation and/or viability [57,58]. Here, the myocyte-C2C12-EVs were labelled with PKH26 (red; Figure S5A), and assessed for % labelling by fluorescent NTA (Figure S5B) and for effect on EV size by NTA (Figure S5C). The PKH26-labelled myocyte C2C12-EVs (50 ng/mL; Figure S5D) were then added to the PKH67-labelled CMT 64/61 cells and monitored for cellular uptake.

**Figure 3:**
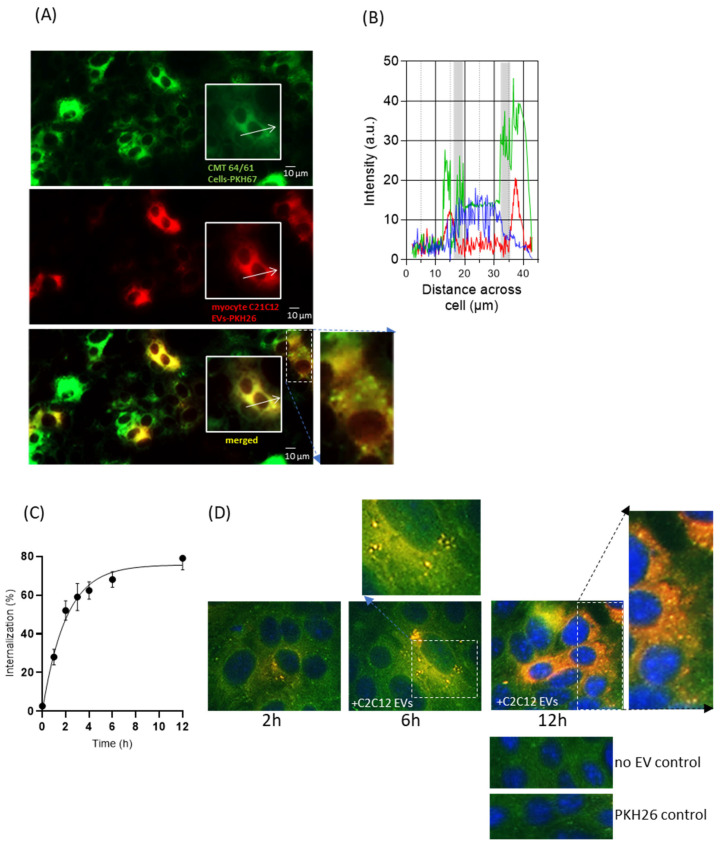
Uptake of PKH26-labelled C2C12-EVs by CMT 64/61 lung carcinoma cells is a time-dependent and selective process. (**A**) A representative fluorescent microscopy image of PKH67-labelled lung carcinoma cells (CMT 64/61) (green) also showing the internalised PKH26-labelled C2C12-EVs (red) after 24 h incubation with the lung carcinoma cell line CMT 64/61 (green) and the overlap of PKH dyes (yellow). Scale bar = 10 µm The white arrows in (**A**) indicate the line-scan fluorescence intensities for PKH67 (green), PKH26 (red) and DAPI (blue) which in (**B**) are measured along these white arrows. The PKH26 C2C12-EVs have taken up a perinuclear location in the CMT 64/61 cells; the grey regions indicate the likely location of the nuclear membrane, (**C**). Having marked an area of the plate and photographed it for up to 12 h, a time course for internalisation of PKH26-labelled C2C12-EVs within CMT 64/61 cells was obtained. To remove surface EVs not internalised, an acid wash using glycine (low-pH buffer) was performed. All values are expressed as the mean ± SD of three independent experiments. The uptake data was fitted to a one-phase exponential association curve using GraphPad Prism (version 6.1). (**D**), Representative fluorescent micrographs showing overlay of PKH67 (green)-labelled lung carcinoma cells co-cultured with PKH26 (red)-labelled myocyte EVs for 2, 6 and 12 h, and absence-of-EV and PKH26 control. DAPI staining (blue) indicates cell nuclei. An increased uptake of EVs was detected by selected carcinoma cells.

Figure 3A shows internalisation of the C2C12 myocyte EVs by the CMT 64/61 cells. The outlined inset images in 3A were assessed for relative fluorescence distribution profiles for PKH67 and PKH26 signals, as shown in Figure 3B; this indicates that the EVs were taken up in a perinuclear distribution (Figure 3A,B). The uptake of the C2C12-EVs was also shown to be time-dependent (Figure 3C,D). When looking at a select field every 60 min, by 12 h a 79% uptake of C2C12-EVs was confirmed in the CMT 64/61 cells (Figure 3C). PKH26-labelled EVs observed as distributed on the cell surface within 2 h, were internalised by 6 h and were visible as punctate fluorescence signals in the cell cytoplasm, with the signal intensity increasing with time up to a 12 h time point. As well as an increased uptake with time, in initial experiments, the EV internalisation seemed to be selective for certain cells, possibly explaining the morphological changes observed earlier in only some cells (Figure 3D).

#### 3.4.3. C2C12-EVs Exert a Cytotoxic Effect on Lung Carcinoma (CMT 64/61) Cells

**Figure 4:**
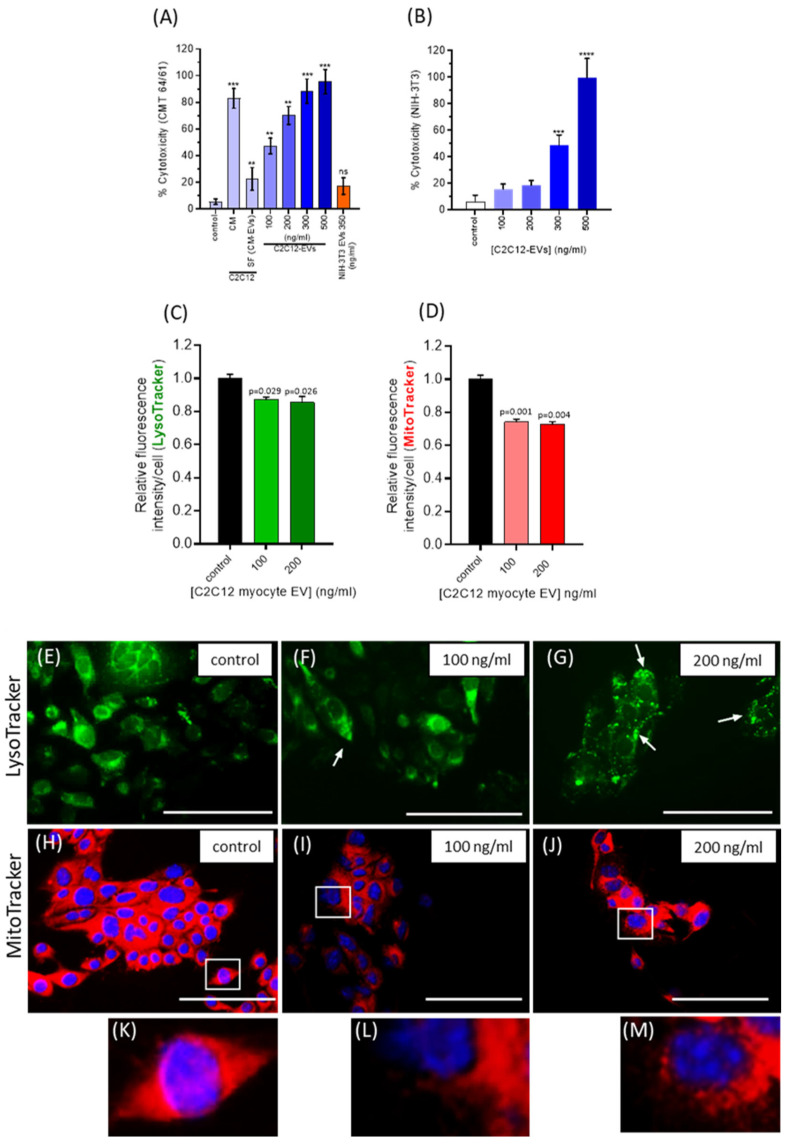
C2C12-EVs induce selective cytotoxicity and lysosomal and mitochondrial dysfunction in highly metastatic lung carcinoma cells. (**A**) Lung carcinoma cells (CMT 64/61) were treated with increasing concentrations of C2C12-EVs. Cell culture supernatant was collected after 24 h and a cytotoxicity assay performed. A significant dose-dependent cytotoxic effect was noted in cells treated with EVs at between 100 and 500 ng/mL. (**B**) The % cytotoxicity of fibroblast (NIH-3T3) cells treated with different concentrations of C2C12-EVs in contrast showed no significant change in cytotoxicity with 100 ng/mL or 200 ng/mL C2C12-EVs. Data represent the mean ± SD of the three independent experiments; *p* ≤ 0.01 (**); *p* ≤ 0.001 (***); *p* ≤0.0001 (****) were considered statistically significant; ns = non significant. (**C**) CMT 64/61 murine lung carcinoma cells were treated with C2C12-EVs for 24 h and stained with LysoTracker Green DND-26 or MitoTracker Red CMXRos for 30 min. Fluorescence intensity decreased with increasing concentration of C2C12-EVs. In both (**C**) and (**D**) fluorescence intensity decreased with added C2C12-EVs. In both, *p* ≤ 0.05 was considered significant (values are the mean ± SD of three independent experiments. In (**E**–**M**) similarly treated CMT 64/61 cells were observed by fluorescent microscopy (Scale bar, 100 µm in representative images). Compared to control, untreated cells (**E**), lysosomes appeared larger in cells treated with 100 ng/mL of EVs, and particularly more distinct with 200 ng/mL of EVs, as indicated by white arrows (**F**,**G**). Fluorescent micrographs of CMT 64/61 cells treated with C2C12-EVs showed mitochondrial changes (**I**,**J**) and zoomed-in images (**L**,**M**) indicated mitochondrial fragmentation with increasing C2C12-EV concentration and swelling, particularly with the higher dose of myocyte EVs (200 ng/mL) (J/M).

Having shown C2C12-EVs to dose-dependently limit the CMT 64/61 viability and migration, they were next investigated for cytotoxicity of CMT 64/61 cells. The Promega CytoTox 96 cytotoxicity assay was used to assess the release of lactate dehydrogenase [59]. CMT 64/61 cells, seeded at 1 × 10^4^ cells/well, in triplicate, in 5% EV-depleted FBS were treated with C2C12-EVs. After 24 h of treatment with 100 and 200 ng/mL C2C12-EVs, the CMT 64/61 cells showed 47% and 70% cytotoxicity, respectively (Figure 4A). In comparison, the NIH-3T3 fibroblasts showed only 16% and 18% cytotoxicity (at 100 and 200 ng/mL EVs, respectively) (Figure 4B). However, when applying 500 ng/mL myocyte EVs, high levels of cytotoxicity of NIH-3T3 cells were observed as well as for CMT 64/61 cells. As 100 ng/mL and 200 ng/mL myocyte EVs were non-toxic to the normal NIH-3T3 cells, these concentrations were used in further experiments. NIH-3T3 EVs (350 ng/mL) showed a non-significant increase in cytotoxicity compared to untreated control.

#### 3.4.4. C2C12-Derived EVs Induce Lysosomal and Mitochondrial Changes in Lung Carcinoma (CMT 64/61) Cells

We hypothesised that the C2C12-EV-induced cytotoxicity of CMT 64/61 cells might be associated with changes in cell membrane permeability, including that of the lysosomal membrane. This would release lysosomal proteases into the cytosol [60], in turn leading to protein degradation by hydrolytic enzymes and apoptosis [60,61,62,63]. To further investigate, a LysoTracker Green DND-26 assay was performed. The CMT 64/61 cells (1 × 10^4^ per well of a chamber slide) were treated with C2C12-EVs for 24 h and incubated for 45 min with 75 nM LysoTracker in growth medium (with 5% EV-depleted FBS) and lysosome fluorescent intensity measured. The results showed that after adding C2C12-EVs, LysoTracker green intensity in the CMT 64/61 cells was significantly reduced (Figure 4C), indicative of reduced numbers of lysosomes. Compared to control, untreated cells (Figure 4E), LysoTracker staining indicated that lysosomes appeared larger and more distinct in the C2C12-EV-treated cells (white arrows in Figure 4F,G).

As lysosomal functions regulate mitochondrial activities [64,65], MitoTracker Red CMXRos fluorescent dye was used to monitor mitochondrial changes in CMT 64/61 lung carcinoma cells (1 × 10^4^ per well), treated with C2C12-EVs in the presence of 5% EV-depleted FBS for 24h. Fluorescent intensity (excitation/emission maxima, 579/599 nm) of CMT 64/61 cells, stained with MitoTracker, decreased significantly following addition of C2C12-EVs (100 and 200 ng/mL, respectively, Figure 4D), indicating a decrease in mitochondrial membrane potential, a hallmark of mitochondrial dysfunction. Fluorescent microscopy showed mitochondrial changes in the treated CMT 64/61 cells compared to control, untreated cells (Figure 4H–J; zoomed-in images in Figure 4K, L and M, respectively). In the myocyte EV-treated CMT 64/61 cells, the mitochondria showed fragmentation (Figure 4I and inset), and those cells treated with the higher dose of 200 ng/mL C2C12-EVs showed additional signs of swelling (Figure 4J and inset (Figure 4M)). It should be noted that these observations on mitochondrial and lysosomal dysfunction do not distinguish from C2C12-EVs directly inducing these effects on CMT 64/61 cells or that they are a consequence of inducing apoptosis, where mitochondrial damage perhaps through ROS release then leads to lysosomal impairment.

#### 3.4.5. C2C12-EVs Induce the Mitochondrial-Mediated Intrinsic Pathway of Apoptosis in Lung Carcinoma Cells (CMT 64/61)

**Figure 5:**
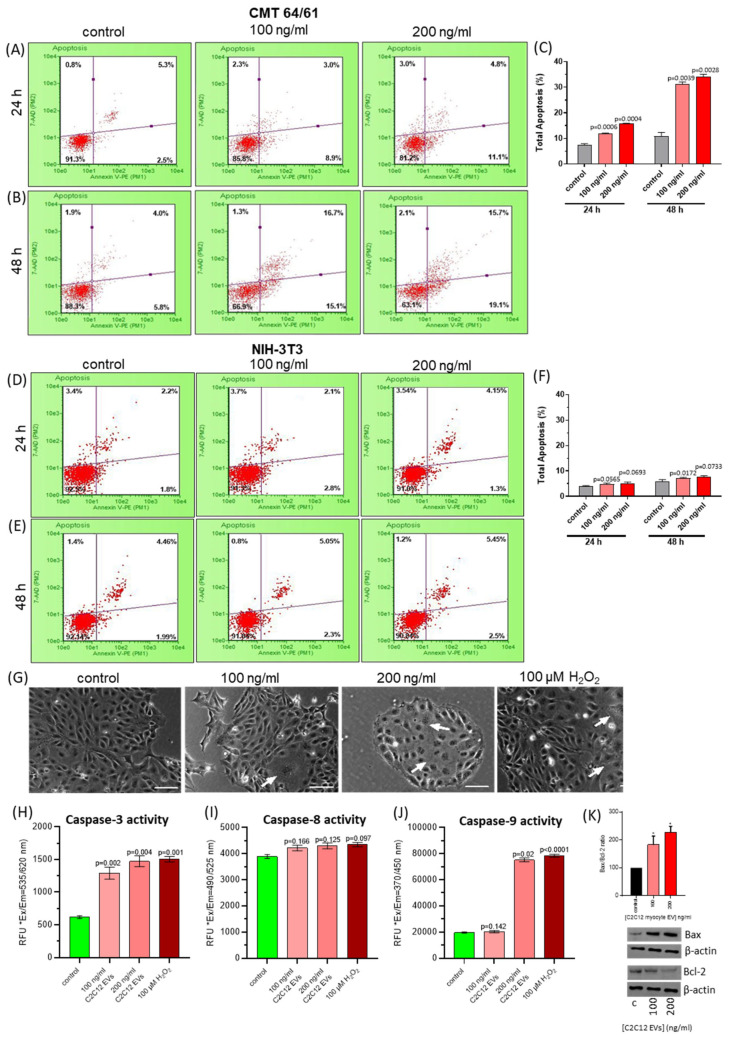
C2C12-EVs dose-dependently induce apoptosis and caspase 3 and 9 activity in highly metastatic lung carcinoma cells (CMT 64/61). CMT 64/61 cells were treated with C2C12-EVs (100 or 200 ng/mL) for 24 h (**A**) and 48 h (**B**) and analysed for apoptosis by flow cytometry using Guava Nexin Reagent. The dot plots show lower-left quadrant: viable cells, [Annexin V-PE^−^ and 7-AAD^−^], lower-right quadrant: cells in the early stages of apoptosis [Annexin V-PE^+^ and 7-AAD^−^], upper-right quadrant: cells in the late stages of apoptosis or dead (by necrotic or apoptotic mechanisms) [Annexin V-PE^+^ and 7-AAD^+^] and upper-left quadrant: mostly nuclear debris [Annexin V-PE^−^ and 7-AAD^+^]. The results in A and B are presented as a bar chart in (**C**), indicating the percentage of total apoptosis (early plus late) analysed by the Guava Nexin software, version 2.7; *p* ≤ 0.05 was considered significant. Compared to control cell cultures, a significant percentage of apoptosis was observed by 24 h in myocyte EV-treated carcinoma cells indicating both dose- and time-dependent increases. Murine fibroblast (NIH-3T3) cells were treated with different concentrations of C2C12-EVs for 24 h (**D**) and 48 h (**E**) and data obtained by the Guava Nexin assay was presented as dot plots and summarised as a bar chart showing % total apoptosis in (**F**); *p* ≤ 0.05 was considered significant. Compared to the untreated control cells, no significant difference was noted in the cultures of NIH-3T3 cells treated with C2C12-EVs. In (**G**), increasing concentrations of myocyte EVs induced morphological changes in CMT 64/61 carcinoma cells after 24 h, indicating a dose-dependent change in cell membrane asymmetry, indicated by white arrows. Apoptosis-inducing H_2_O_2_ was used as a positive control, these carcinoma cells showing a similar cell membrane asymmetry pattern; Bar, 100 μm. In (**H**–**J**) the caspase activity of C2C12-EV-treated CMT 64/61 carcinoma cells, after 48 h, is demonstrated for caspase 3, 8 and 9, respectively. The results (in **C**,**D**,**H**–**J**) were expressed as the mean ± S.D. (*p* ≤ 0.05 vs. control group, from three independent experiments, n = 3). (**K**) The Bax/Bcl-2 ratio in CMT 64/61 cells treated with 100 and 200 ng/mL C2C12-EVs was calculated from the protein expression levels determined using scanning densitometry; *p* < 0.05 (*) was considered statistically significant (Original Western blotting images are available in the Supplementary Material).

Further to the observed mitochondrial dysfunction in CMT 64/61 cells treated with C2C12-EVs, we investigated effects on apoptosis. CMT 64/61 (seeded at 1 × 10^4^ cells/well in 24-well plates) were treated with C2C12-EVs, and incubated for 24 and 48 h. Cells were then detached, washed and treated with 1% EV-depleted FBS and 100 μL Nexin reagent for 20 min/RT, and assessed by flow cytometry for percentage non-apoptotic cells (AnV−/7-AAD−), early apoptotic cells (AnV+/7-AAD−), or late-stage apoptotic and dead cells (AnV+/7-AAD+). Following 24 h, the percentage of live CMT 64/61 cells treated with the 100 and 200 ng/mL myocyte EV doses had decreased significantly in a dose-dependent manner, from 91.3% (untreated control sample) to 85.8% and 81.2%, respectively. Furthermore, the percentage of early apoptotic CMT 64/61 cells in 100 and 200 ng/mL C2C12-EV-treated samples had increased 3.6- and 4.4-fold (8.9% and 11.1%, respectively, over untreated control (2.5%)). A similar pattern was observed for the percentage of CMT 64/61 cells in late apoptosis (Figure 5A). By 48 h, the live CMT 64/61 cell population had decreased significantly to 66.9% in 100 ng/mL C2C12-EV-treated and 63.1% in 200 ng/mL C2C12-EV-treated cell cultures. This correlated to a significant increase in late apoptosis in the C2C12-EV-treated CMT 64/61 cells from 4% (untreated control) to 16.7% and 15.7% for 100 and 200 ng/mL C2C12-EV-treated samples, respectively. Compared with the 24 h late apoptotic CMT 64/61 cell population, there was a ~5.6-fold increase in the 100 ng/mL C2C12-EV-treated cells and a ~3.3-fold increase in the 200 ng/mL C2C12-EV-treated cells. Similarly, a ~1.7-fold increase in early apoptosis occurred in both C2C12-EV-treated CMT 64/61 cell samples by 48 h (compared to that at 24 h). These results support a dose-dependent increase in apoptosis (decrease in cell viability) and increase in cytotoxicity (late apoptosis) at 24 h, following treatment of CMT 64/61 carcinoma cells with C2C12-EVs. By way of control, there were only marginal increases in apoptosis when normal NIH-3T3 fibroblasts were treated with C2C12-EVs at both 24 and 48 h (Figure 5D–F).

To identify the apoptotic pathway triggered in the CMT 64/61 cells by the C2C12-EVs, caspase 3, 8 and 9 detection assays were used. Cells (1 × 10^4^/well in 96-well plates) were treated with C2C12-EVs (100 and 200 ng/mL) for 24 h at 37 °C. Whilst low concentrations of H_2_O_2_ induce the intrinsic apoptotic pathway [66] and higher concentrations induce necrotic cell death [67], as a positive control the CMT 64/61 carcinoma cells were therefore treated with 100 µM of H_2_O_2_ for 24 h. Fluorescent intensity was then measured in all samples. CMT 64/61 cell numbers were reduced with increasing concentration (100 or 200 ng/mL) of C2C12-EVs (Figure 5G). Caspase 3 activity was dose-dependently increased in myocyte C2C12-EV-treated CMT 64/61 cells (Figure 5H). There was no significant change, however, in caspase 8 activity (Figure 5I). Caspase 9 activity was significantly increased only in cells treated with the 200 ng/mL dose of C2C12-EVs (Figure 5J). Caspase 3 is common to both the extrinsic and intrinsic apoptotic pathways, whilst caspase 8 and caspase 9 are specific to the extrinsic and intrinsic apoptotic pathways, respectively. As a significant increase in caspase 3 and 9 was observed in the CMT 64/61 carcinoma cells treated with 200 ng/mL C2C12-EVs, this suggests that the C2C12-EVs induced the mitochondrial-mediated, intrinsic pathway of apoptosis. This was further supported by a dose-dependent increase in Bax/Bcl-2 ratio with increasing C2C12-EVs (Figure 5K).

### 3.5. C2C12-EVs Exert S Phase Cell Cycle Arrest on Lung Carcinoma Cells (CMT 64/61)

**Figure 6:**
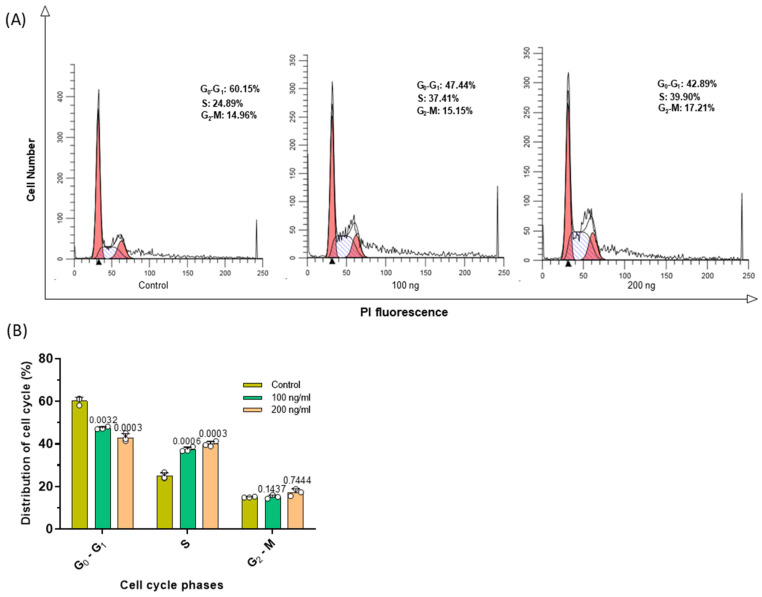
C2C12 myocyte EVs cause S phase cell cycle arrest in lung carcinoma cells (CMT 64/61). Carcinoma cells treated with or without C2C12-EVs (100 ng/mL and 200 ng/mL) for 48 h were stained with Guava cell cycle reagent and flow cytometry used to assess the cell cycle distribution. (**A**) The histograms show the data analysed by ModFit software. Red histogram, G0/G1; hashed histogram, S and clear histogram, G2/M. (**B**) The percentage distribution in the cell cycle as indicated in the histograms in (**A**) is here shown in a bar chart as mean ± S.D. from three independent experiments (*p* ≤ 0.05 vs. control group); *p* ≤ 0.05 was considered significant.

As the C2C12-EVs induced apoptosis in the CMT 64/61 lung carcinoma cells, their effect on cell division was then investigated by monitoring cell cycle events using the nuclear DNA stain, propidium iodide (Guava^®^ Cell Cycle Reagent). Stained CMT 64/61 cells treated as before with the C2C12-EVs were examined by flow cytometry and analysed by ModFit software (Figure 6A). This showed the C2C12-EVs to dose-dependently induce cell cycle arrest of the CMT 64/61 cells in S phase, where DNA replication occurs, having progressed from G0/G1, thereby preventing the cells from entering the proliferative phase. Compared to control, untreated cells, where there was 24.9% of cells in S phase, for the CMT 64/61 cells treated with 100 and 200 ng/mL C2C12-EVs, the percentage cells in S phase increased to 37.4% and 39.9%, respectively (Figure 6B). S phase arrest of CMT 64/61 cells treated with C2C12-EVs therefore prevented DNA replication. This is consistent with these cells moving more slowly through G2/M than their untreated control counterparts. Hence, there was a decrease in percentage of cells in G0/G1, from 60.1% for control to 47.4% and 42.9% for 100 and 200 ng/mL, respectively, of C2C12-EV-treated CMT 64/61 cells.

### 3.6. EV Protein Cargo Analysis of Myocyte (C2C12-) EVs and Fibroblast (NIH-3T3) EVs

To identify possible contributions of EV protein cargoes to EV-mediated changes in cellular function of the treated carcinoma cells, the proteomic EV cargoes of C2C12-derived EVs were assessed and compared to those from EVs of the NIH-3T3 fibroblast cell line. Proteins present in duplicate samples and with mean spectral counts of ≥5 were included for further analysis. Of the 1001 proteins detected in the EVs of both cell types, only 387 contained ≥ 5 peptides and were therefore deemed reliable and included in further analysis. These proteins are presented in Supplementary Table S1. Protein hits unique to myocyte C2C12-EVs are furthermore summarised in Table 1.

#### 3.6.1. Protein–Protein Interaction (PPI) Network Analysis of EV Protein Cargoes from C2C12 Myocytes and NIH-3T3 Fibroblasts

**Figure 7:**
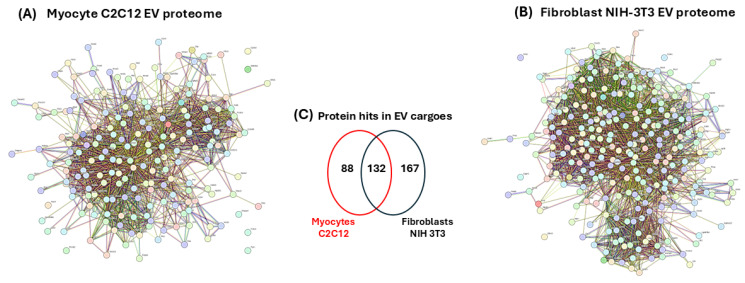

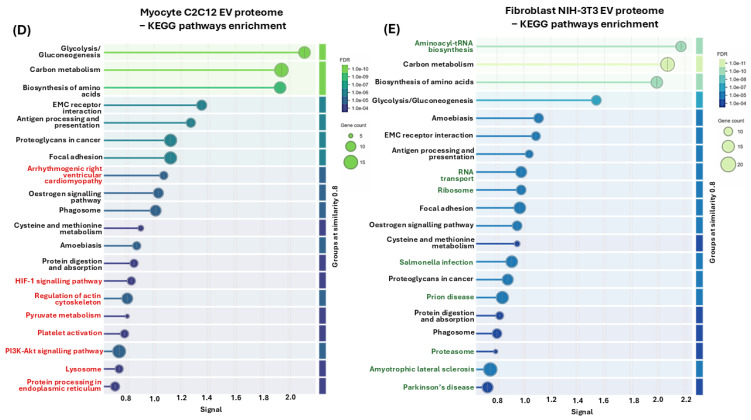
EV proteomes for myocyte and fibroblast EV cargoes. Protein–protein interaction (PPI) networks for (**A**) C2C12-EVs and (**B**) fibroblast NIH-3T3 EVs. (**C**) Venn diagram representing numbers of shared and unique proteins hits identified in the EV cargoes of C2C12- and NIH-3T3-EVs, respectively. KEGG pathways for EV proteomes from myocyte (**D**) and fibroblast (**E**) EVs. Top 20 KEGG pathways are shown. Pathways unique to the myocyte EV proteomes are highlighted in red, while those unique to the fibroblast EV proteomes are highlighted in green.

To identify the protein–protein interaction networks of the myocyte and fibroblast EV cargoes, the data represented in Supplementary Table S1 were submitted to STRING analysis (https://string-db.org/, accessed on 19 March 2021). The resulting protein–protein interaction networks for EV cargoes of both cell lines are presented in Figure 7A,B. The *p*-values for the protein–protein interaction (PPI) networks were found to be *p* < 1.0 × 10^−16^, indicating that the proteins have more interactions amongst themselves than would be expected for a similarly sized, random group of proteins drawn from the genome. Of the 387 proteins which were included in the analysis, 86 were specific to the C2C12-EVs and 167 were specific to the fibroblast-EVs, while 132 proteins were common targets to both EV populations (Figure 7C). For the identification of differences between the EV cargoes of the myocyte and fibroblast (control) cell lines, functional enrichment pathway analysis was carried out in STRING. This revealed 50 KEGG pathways associated with the myocyte EV proteome, and 42 KEGG pathways associated with the fibroblast EV proteome, respectively. Figure 7D,E list the top 20 KEGG pathways for the EV proteomes of both cell lines, respectively. For the myocyte EV proteomes, unique KEGG pathways out of these top 20 were arrhythmogenic right ventricular cardiomyopathy, HIF-1 signalling pathway, regulation of actin cytoskeleton, pyruvate metabolism, platelet activation, PI3K-Akt signalling pathway, lysosome and protein processing in ER (Figure 7D). KEGG pathways unique to the fibroblast proteome (out of the top 20 KEGG pathways) were aminoacyl-tRNA biosynthesis, RNA transport, ribosome, salmonella infection, prion disease, proteasome, ALS, Parkinson’s disease (Figure 7E). Additional shared KEGG pathways for the EV proteomes of both myocytes and fibroblasts were glycolysis/gluconeogenesis, carbon metabolism, biosynthesis of amino acids, EMC receptor interaction, antigen processing and presentation, proteoglycans in cancer, focal adhesion, oestrogen signalling pathway, phagosome, cysteine and methionine metabolism, amoebiasis, protein digestion and absorption.

#### 3.6.2. Protein–Protein Interaction (PPI) Network Analysis of EV Protein Cargoes Specific to Only C2C12 Myocytes or NIH-3T3 Fibroblasts

**Figure 8:**
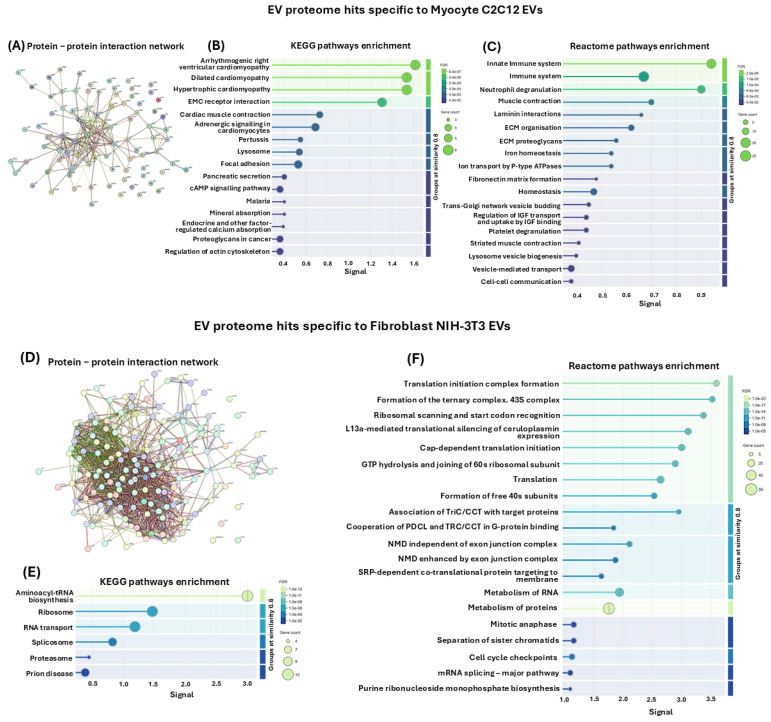
(**A**) PPI network for EV proteome hits unique to the C2C12-EVs, and not shared with the NIH-3T3-EVs. (**B**) Associated KEGG pathways. (**C**) Associated Reactome pathways. (**D**), PPI network for EV proteome hits unique to the fibroblast NIH-3T3-EVs, and not shared with the C2C12-EVs. (**E**) Associated KEGG pathways. (**F**) Associated Reactome pathways.

In addition to analysing PPI networks and associated KEGG pathways of all proteins identified in EVs of both myocytes and fibroblasts, the same analysis was carried out for the protein hits unique to either myocyte C2C12-EVs or fibroblast NIH-3T3-EVs, respectively. A PPI network was generated for the myocyte EV-specific protein hits (Figure 8A), revealing 16 associated KEGG pathways (Figure 8B) and 18 associated Reactome pathways (Figure 8C). The KEGG pathways identified were arrhythmogenic right ventricular cardiomyopathy, dilated cardiomyopathy, hypertrophic cardiomyopathy, EMC receptor interaction, cardiac muscle contraction, adrenergic signalling in cardiomyocytes, pertussis, lysosome, focal adhesion, pancreatic secretion, cAMP signalling pathway, malaria, mineral absorption, endocrine and other factor-regulated calcium absorption, roteoglycans in cancer, regulation of actin cytoskeleton (Figure 8B). The associated Reactome pathways were innate immune system, immune system, neutrophil degranulation, muscle contraction, laminin interactions, extracellular matrix organisation, ECM proteoglycans, iron homeostasis, ion transport by P-type ATPases, fibronectin matrix formation, homeostasis, Trans-Golgi network vesicle budding, regulation of IGF transport and uptake by IGF binding, platelet degranulation, striated muscle contraction, lysosome vesicle biogenesis, vesicle-mediated transport, cell–cell communication (Figure 8C).

In comparison, PPI networks for protein hits identified as specific to the fibroblast NIH-3T3 cells, and not shared with the C2C12-EVs (Figure 8D), revealed six unique associated KEGG pathways, which were aminoacyl-tRNA biosynthesis, ribosome, RNA transport, splicosome, proteasome and prion disease (Figure 8E). The top 20 associated Reactome pathways are furthermore shown (Figure 8F) as follows: translation initiation complex formation, formation of the ternary complex. 43S complex, ribosomal scanning and start codon recognition, L13a-mediated translational silencing of ceruloplasmin expression, cap-dependent translation initiation, GTP hydrolysis and joining of 60s ribosomal subunit, translation, formation of free 40 s subunits, association of TriC/CCT with target proteins, cooperation of PDCL and TRC/CCT in G-protein binding, NMD independent of exon junction complex, NMD enhanced by exon junction complex, SRP-dependent co-translational protein-targeting to membrane, metabolism of RNA, metabolism of proteins, mitotic anaphase, separation of sister chromatids, cell cycle check points, mRNA splicing—major pathway, purine ribonucleoside monophosphate biosynthesis (Figure 8F).

#### 3.6.3. Significantly Enriched or Diminished C2C12-Derived EV Protein Cargo Hits

**Figure 9:**
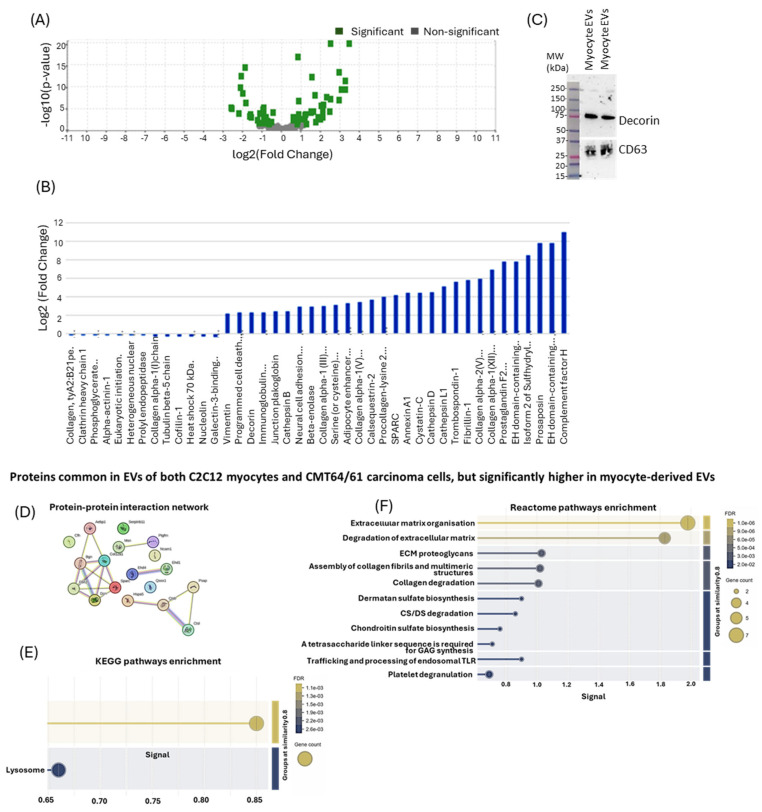
Significantly diminished or enriched proteins in EV cargoes of C2C12-EVs. (**A**) Volcano plot highlighting significantly modified proteins. (**B**) There were 13 proteins identified as significantly diminished and 29 proteins as significantly enriched (including decorin). (**C**) Decorin was further verified in myocyte-EVs by Western blotting; positive CD63 staining was included as an EV-specific marker. Top protein hits common to myocyte and fibroblast cells, which were significantly elevated in the myocyte-EVs (Table 2) (Original Western blotting images are available in the Supplementary Material). (**D**) PPI network; (**E**) KEGG pathways enrichment analysis; (**F**) Reactome pathways enrichment analysis.

For further analysis, the 86 C2C12-EV-specific proteins which were identified were verified in UniProt (https://www.uniprot.org/, accessed on 19 March 2021) to obtain UniProt names, protein accession numbers and Gene symbols (Table 1). The inbuilt Scaffold 4.11.1 statistical tool was used to calculate the fold change by category for all the C2C12-specific EV proteins (using fibroblast as the referenced category). Based on two replicates, Fisher’s Exact Test and multiple test correction (Benjamini–Hochberg correction) were selected; considering two-fold changes in enriched or diminished proteins with *p*-values ≤ 0.05. The fold change cut-off points were computed by the Scaffold software, revealing a total of 220 myocyte proteins, 29 or which were enriched and 13 diminished (Figure 9A,B). Of the 29 enriched proteins, only two are described as inhibiting cancer cell growth and migration, as well as causing lysosomal/mitochondrial dysfunction and increasing apoptosis: cystatin C (through its inhibition of cathepsins) [68] and decorin [69]. For the purposes of this study, we only validated decorin expression in myocyte EVs by Western blotting (Figure 9C), while CD63 was used as a loading control and EV-specific marker. The Western blot confirmed the strong presence of decorin in myocyte EVs from the proteomic analysis.

From 132 proteins common to both cell types, built-in *t*-test analysis identified those proteins (*p* < 0.05) significantly highly expressed in C2C12-EVs. These 19 C2C12-EV proteins are listed in Table 2.

To identify their protein–protein interaction (PPI) network, STRING analysis (https://string-db.org/) was carried out (Figure 9D) and associated KEGG pathways (Figure 9E) and Reactome pathway (Figure 9F) enrichment analysis was performed. This revealed enrichment in two KEGG pathways for this protein set: antigen processing and presentation, and the lysosome pathway (Figure 9F). There were 11 Reactome pathways associated with this protein set: extracellular matrix organisation, degradation of ECM, ECM proteoglycans, assembly of collagen fibrils and multimeric structures, collagen degradation, dermatan sulfate biosynthesis, CS/DS degradation, chondroitin sulfate biosynthesis, GAG synthesis, trafficking and processing of endosomal TLR, and platelet degranulation (Figure 9F).

## 4. Discussion

Extracellular vesicles (EVs) play significant roles in physiological and disease processes, including infectious, metabolic and neurodegenerative diseases, cancer and in ageing [70,71,72]. EVs modulate these processes by transferring their cargo of proteins, lipids and nucleic acids to recipient cells. Therefore, modulation of EV-mediated cargo is important in the context of disease processes. With their wide-ranging functions, a focus has also been on clinical applications and their potential as diagnostic [73,74] and therapeutic agents [70,74,75]. Cancer progression in skeletal muscle is very rare and mechanisms underlying this are poorly understood, highlighting the need to investigate possible roles for EV-mediated effects. The current study therefore used murine in vitro cell models to assess potential anti-cancer effects of C2C12-EVs on metastatic lung cancer cells.

### 4.1. Myocyte EV Isolation and Characterisation

The current study adhered to the ISEV guidelines (MISEV 2018, 2024; [46,47]) for EV characterisation, and utilised differential centrifugation to isolate small EVs (sEVs), with a mean size value of < 200 nm, followed by further characterisation by nanoparticle tracking analysis, dynamic light scattering, transmission electron microscopy and surface marker detection. The yield of sEVs from myocyte cells was approximately 8.81 ×10^10^ ± 2.74 × 10^8^ particles/mL from 1 × 10^8^ myocyte cells within a 24 h time span. The presence of two EV-specific markers, CD63 and TSG101 [76,77], but not of the contaminant marker GM130, was confirmed.

### 4.2. Lung Carcinoma Cells Selectively Govern Myocyte EV Uptake

EVs are known to alter physiological and pathological states of recipient cells by inducing intracellular signals and altering molecular pathways. This is achieved by either binding with recipient cell surface receptors or internalisation and release of the EV intravesicular cargo into the recipient cell’s cytosol [57,78]. Interestingly, skeletal muscle-derived EVs injected intraperitoneally in mice have been shown to be taken up by various organs including lung [79], raising the potential that they could be used therapeutically [80]. Despite many recent advances in the EV field, the exact mechanism of EV uptake remains unclear. Initially, many studies have reported EV uptake to occur in any cell [81,82] but more recent studies indicate that it is a highly specific process, as the recipient cells and the EVs require the correct surface receptors and ligands for interaction [83,84,85,86,87]. The observations in our study aligned with the latter process, as only few carcinoma cells in a colony were found to take up the applied C2C12-EVs (“EV transplant”). A time-dependent uptake was observed, resulting in higher fluorescent intensities in certain carcinoma cells, possibly due to differing degrees of uptake by particular cells within the same colony. This could be due to EV uptake being affected by the metabolic status of recipient cells [86]. The cell cycle phase of the recipient cells may also affect uptake, as with prostate cancer cells found to have increased EV uptake during G2/M phase and less in G0/G1 or S phase [87]. Using colorectal cancer cells, it was found that EVs from hypoxic cells were more readily taken up than normoxic EVs [88]. Additional mechanisms of EV uptake, such as endocytosis, must also be considered including receptor-mediated endocytosis, macropinocytosis and phagocytosis. The differences in cellular EV uptake mechanisms may furthermore be dependent on the cell type, and EVs may also be simultaneously taken up into cells by different mechanisms [89]. Another consideration is that the observed effects might simply be due to the tropism of C2C12 EVs for lung cancer cells due to their corona (hard and soft) and EV membrane.

### 4.3. Anti-Tumourigenic Effects of Skeletal Muscle-Derived EVs

Next, we investigated whether skeletal muscle-derived EVs play a role in suppressing cancer cells. Most organs and tissues are subjected to metastasis with the striking exception of skeletal muscle, in which cancer metastasis is very rare [23,24,25,26,90]. This is surprising as it is a highly vascularized tissue and compromises ~50% of the body mass. Although several studies have linked the rarity of cancer progression in skeletal muscle to high levels of lactic acid [31], low molecular weight factors [37] and many cytokines [91,92,93], further mechanisms remain to be elucidated including the role of EVs. Our preliminary observations suggested that highly metastatic murine lung carcinoma cells (CMT 64/61) failed to colonise skeletal muscle cells (C2C12). However, this will require further research as the differentiation medium for C2C12 was withdrawn and the co-culture was carried out in 2% (EV-free) FBS. Comparing our data with in vivo findings in mice, following tail vein injection of carcinoma cells, there is a lack of tumour appearance in skeletal muscle [34,55]. Conversely, direct injection into the hindlimb muscles of mice is a standard method that allows for the establishment of tumours. We believe that tail injection fails because of the unfavourable microenvironment in skeletal muscle for disseminated tumour cells. Whilst in vivo there are many inhibitory factors including immunosurveillance, metabolic barriers and high metabolic stress, the unfavourable environment in our experiments, we hypothesise, is due to the C2C12-EVs, reducing the ability of the (migrating) carcinoma cells to attach, as seen by the decreased number of cell colonies established and by the reduced number of migrated CMT 64/61 cells. However, direct injection of cancer cells in vivo does enable their establishment in skeletal muscle as they bypass normal barriers (met in the circulation) and the local microenvironment. Directly injected cells can also interact with muscle fibres, promoting tumour invasion, rather than inhibiting it.

Our findings therefore align with growing evidence, identifying EVs as a pivotal constituent of the cellular microenvironment. Recently there has been increasing interest in the potential role of EVs as a positive and negative modulator of the tumour microenvironment (TME) for cancer progression [94]. For example, EVs from cancer cells were found to interact with immune cells to modulate the microenvironment and enhance immune evasion, cancer growth and progression [95]. By contrast, EVs derived from certain cell types are also known to exert inhibitory effects on cancer cells, including dendritic cell-derived EVs which were able to modulate the TME and enhance immune responses to reduce lung metastasis [96]. Mesenchymal stem cell-derived EVs, for example, also suppress human breast cancer angiogenesis [97].

In addition to examining direct effects of EVs isolated from the myocyte culture medium, the conditioned culture medium was also assessed. Steven Paget’s “Seed and Soil” hypothesis for metastasis describes the need of a receptive microenvironment or niche for disseminating carcinoma cells to engraft distant sites [98,99]. In alignment with this, we found that the skeletal muscle microenvironment in the form of skeletal muscle-conditioned media exerts inhibitory effects on carcinoma cell viability in culture. Other studies have also observed similar effects exerted by skeletal muscle-conditioned media, including on carcinoma cell apoptosis [38] and growth inhibition [37]. Our data did suggest though that the anti-cancer effects of myocyte EVs on carcinoma cells were comparable to those mediated by the myocyte-conditioned medium.

These findings supported roles for skeletal muscle-derived EVs as a local tumour suppressor. The lung carcinoma cells treated with the C2C12-EVs also showed a dose-dependent decrease in cell viability and a significant reduction in number of cell colonies formed, as well as of reduced size. The C2C12-EVs may therefore be promoting an unfavourable environment for migrating carcinoma cells, lowering their capacity for attachment, as reflected in the low number of cell colonies established. Furthermore, changes in cell morphology, including irregular shape and loss of cell membrane integrity, were noted in some of the lung carcinoma cells treated with the C2C12-EVs.

#### 4.3.1. Role of C2C12-EVs in Inducing Apoptosis of Carcinoma Cells

The potential of EVs as a biological anti-cancer agent is appealing, considering that optimally it should destroy or incapacitate cancer cells, while causing minimal damage to surrounding healthy bystander cells [75,100,101]. We observed a selective cytotoxicity of the myocyte EVs on the metastatic lung carcinoma cells. The C2C12-EVs, even at comparatively low concentration (100 ng/mL), exerted specific and significant cytotoxicity on the carcinoma cells but not on normal (control) fibroblast cells, although at concentrations above 200 ng/mL they did show cytotoxicity to normal cells. This highlights that effective low therapeutic doses of myocyte EVs on cancer cells would need optimisation if to be used therapeutically, to avoid effects on bystander cells. The cytotoxic effects of myocyte EVs on the cancer cells were further assessed as cells’ viability can be impacted by cytotoxicity in various ways, including necrosis and apoptosis, especially as the observed loss of cell membrane integrity and shape indicated such processes. As growing evidence suggests that lysosomes are involved in shaping cell death in a process known as lysosome-dependent cell death (LDCD) [102], this was further assessed. Our results indicated a dose-dependent decrease in the numbers of lysosomes, accompanied by an increase in lysosome size, in response to incubation of lung carcinoma cells with the C2C12-EVs. Increased lysosomal membrane permeability has been shown to cause enlargement of lysosomes, which can rupture and release hydrolytic enzymes into the cytosol [103]. This may explain the observed reduction in lysosome numbers observed in the current study. Lysosomes contain high levels of hydrolytic enzymes. In the event of membrane damage, these hydrolytic enzymes are released into the cell cytosol and cause indiscriminate degradation of cellular components, inducing cell death by necrosis [60]. It has also been shown that partial lysosomal membrane permeability can induce cell death by apoptosis [61,62,63,104] and this could be a desirable approach for cancer therapy [102].

As changes in lysosomal function induce changes in mitochondrial membrane composition, which impairs mitochondrial functions and induces apoptosis, we also assessed effects on mitochondria following the C2C12-EV transplant. We found that C2C12-EVs exerted a specific apoptotic effect on the lung carcinoma cells, with a pronounced cell death after 24 h, but with no effect on control fibroblasts. Further investigations revealed impaired mitochondrial functions through reduced mitochondrial membrane potential and increased caspase 3 and caspase 9 levels in carcinoma cells treated with the C2C12-EVs, suggesting mitochondrial-dependent apoptosis mediated by the intrinsic pathway. C2C12-EVs (100 ng/mL) induced apoptosis but without an increase in activity of caspase 9 (only achieved at 200 ng/mL EVs). This will require further investigation but may be through caspase-independent cell death (CICD) in which apoptosis is triggered due to permeabilization of the mitochondrial outer membrane and controlled by the Bcl-2 family proteins Bax/Bak [105].

Inducing apoptosis of cancer cells, whilst limiting death of normal cells, is a promising non-surgical cancer therapy [106,107]. EVs from natural killer cells have, for example, been shown to induce targeted apoptosis of various carcinoma cells via activation of the intrinsic pathway [108], which aligns with our findings here, using C2C12-EVs. The intrinsic apoptotic pathway can be activated by a variety of both intracellular and extracellular factors [109], including changes in lysosomal membrane permeability. This would induce cathepsin B and D release into the cytosol and cleave Bid protein [110], which could induce apoptosis by mediating release of cytochrome c from mitochondria into cytosol [111,112] and mitochondrial outer membrane permeabilisation [113]. C2C12-EVs could also exert direct cytotoxicity on the carcinoma cells, leading to altered membrane potential and mitochondrial outer membrane permeabilization independent of Bid [114]. Our results therefore indicate multiple potential cytotoxic cell death mechanisms to lung carcinoma cells mediated by C2C12-EVs, which will require further in-depth investigation.

#### 4.3.2. Role of C2C12-EVs on Carcinoma Cell Proliferation and Cell Migration

Previous studies on anti-cancer effects of EVs have included assessments of inhibition of cancer growth. For example, gastric carcinogenesis was shown to be affected by epithelial cell-derived EVs [115], while in colon cancer, EVs carrying microRNA-375 were associated with cancer cell progression and dissemination [43]. As a potential new EV-based cancer therapy, non-apoptotic forms of regulated cell death (pyroptosis, ferroptosis and necroptosis) are induced in cancer cells by EVs. For example, macrophages or mesenchymal stem cell-derived EVs induce ferroptosis in recipient cancer cells [116]. With respect to previous studies on C2C12-EVs, skeletal muscle progenitor cell-derived EVs were shown to inhibit prostate cancer cell proliferation [117]. In addition, the effect of skeletal muscle-conditioned media on G0/G1 tumour cell growth has been reported [33]. Our current study is though the first to report anti-proliferative effects of C2C12-EVs, specifically with respect to sEVs, showing that lung carcinoma cells treated with C2C12-EVs exited the cell cycle at S phase, in which cells replicate their DNA. As S phase arrest prevents cell progression to G2/M phase, cell proliferation is inhibited.

The effect of C2C12-EVs on carcinoma cell migration was also assessed. For successful metastasis, carcinoma cells must infiltrate adjacent tissue by intravasation, extravasation and then proliferation at a distance site [118]. Chemotaxis and chemokinesis are known to play crucial roles in cancer metastasis. Organ-specific stromal cells can also induce chemotaxis by releasing signalling proteins that attract cancer cells, causing organ-specific metastasis, and EVs have been reported to modulate and enhance this process [119]. However, EVs derived from some cell types inhibit organ-specific metastasis, including dendritic cell-derived EVs, which modulate the TME and enhance immune responses to reduce lung metastasis [96]. The results obtained in our study, using a cell migration assay, also showed a significant reduction in carcinoma cell migration in the presence of C2C12-EVs. In addition, upon migration to skeletal muscle, carcinoma cells undergo apoptosis, which provides a further explanation for the rarity of metastatic tumours in skeletal muscles.

### 4.4. C2C12-EV Proteome Cargo Analysis and Functional Enrichment Pathways—Putative Anti-Cancer Effects

EVs are known to transport a plethora of biomolecules, including lipids, non-coding RNA and proteins, which can be transferred to recipient cells and exert cellular changes. Here, we analysed EV protein cargoes from the C2C12-EVs, in comparison with fibroblast EVs, using liquid chromatography-based mass spectrometry analysis (LC-MS/MS). Functional enrichment pathway analysis revealed considerable differences between the EV proteomes of the two cell lines, with top unique KEGG pathways for C2C12-EVs including the HIF-1 signalling pathway, regulation of actin cytoskeleton, pyruvate metabolism, PI3K-Akt signalling pathway, platelet activation, protein processing in ER and the lysosome pathway. Our findings correlate with those of a recent report on small EV (sEV) analysis from C2C12 myoblasts [120]. The HIF-1 pathway is strongly associated with cancer progression and metastasis [121,122]. Regulation of actin cytoskeleton has multifaceted roles in cancer progression, including effects on migration, mitochondrial structure and EMT [123,124]. The pyruvate pathway has roles in cancer metabolism and progression, including via mitochondrial effects, and links to glycolysis [125,126,127,128]. The PI3K-Akt signalling pathway promotes cell survival, cell growth and cell cycle progression, and has important roles in oncogenic signalling and cancer metabolism [129,130]. Platelet activation plays important roles in tumour communication, including cancer proliferation, metastasis, invasion and angiogenesis, via EVs [131,132]. Endoplasmic reticulum (ER) stress is associated with many cancers, associated with the tumour and its microenvironment, and has important implications for the unfolded protein response in cancer and cell death [133,134]. The lysosomal pathway has critical roles in cellular homeostasis including roles in regulation of cellular metabolism and cell death [135]. In addition, several other pathways, including the glycolysis/gluconeogenesis and proteoglycans in cancer, both of which play roles in cancer progression, were further associated with EV cargoes of both cell types. In relation to the proteoglycan pathway, many proteoglycans promote tumour cell growth, survival, migration and invasion [136]. Decorin was one of the proteins associated with this pathway, and it contains a GAG side chain, which is either chondroitin sulphate or dermatan sulphate [137], by which it arranges adjacent collagen fibrils. Decorin interacts with several growth factors and their cognate receptors, stimulating different signalling pathways, most of which lead to growth suppression, apoptosis and inhibition of tumour angiogenesis [138]. Thrombospondin-1, also a proteoglycan enriched in myocyte EVs, is an extracellular calcium-binding glycoprotein, of which upregulation has been shown to dose-dependently inhibit prostate cancer proliferation, migration and invasion [139]. It may be postulated that decorin may stimulate thrombospondin-1 secretion and suppress angiogenesis [140]. Cathepsin L1, also identified here, cleaves perlecan, a proteoglycan protein which inhibits the growth and invasiveness of cancer cells [141] and of angiogenesis [142].

Furthermore, out of the top 19 significantly enriched proteins identified in myocyte EVs, compared with the fibroblast EVs, STRING analysis highlighted two top KEGG pathways: “Antigen processing and presentation”, which links to tumour immunogenicity and immune evasion [143] and “Lysosome”, which is associated with the experimental outcomes of the study. Identification of the lysosome pathway in the EV proteome links to cell death where many lysosome-related proteins have been identified as key regulators of apoptotic cell death, including cathepsins [144]. In the LC-MS/MS analysis, cathepsin L1, cathepsin B and cathepsin D were all identified as enriched proteins in myocyte EVs. Overexpression of cathepsin B, D and L in the cytosol has been implicated in degradation of Bid, while cleavage of Bid by cathepsin D induces Bax-mediated release of cytochrome c from mitochondria into the cytosol. In turn, cytochrome c initiates caspase 9 activity followed by stimulation of the caspase 3 cascade, which leads to intrinsic apoptosis [145]. This aligns with our experimental data shown here, suggesting that C2C12-EVs induce mitochondrial membrane permeability, leading to intrinsic apoptosis, confirmed by the observed upregulation of caspase 3 and 9. In cancer, the myokine, cathepsin B, is associated with initiation, tumour growth/proliferation, angiogenesis, invasion and metastasis [146], although its overexpression can induce apoptosis of cancer cells [147]. Cathepsins B, D and L, by degrading Bid, cause the release of cytochrome C from mitochondria, which induces intrinsic apoptosis [148]. The upregulation of cathepsin D and L in myocyte EVs (Figure 9B) suggests that these proteins could induce apoptosis of carcinoma cells via the intrinsic pathway. However, they generally promote proliferation to cancer cells as well as their migration and invasion [147,149,150].

### 4.5. Decorin: An Enriched Myocyte EV Cargo Hit—A Myokine with Anti-Cancer Effects

The proteoglycan decorin was identified in the current study as one of the top enriched proteins in C2C12-EVs. It was also associated with the proteoglycan cancer pathway, which was identified as one of the KEGG pathways associated with the EV proteomes. Decorin is a myokine which belongs to the leucine-rich proteoglycan family and is mainly synthesised by fibroblast, smooth muscle cells and stressed vascular endothelial cells. Originally, decorin was known for its high affinity interactions with collagen fibres and the regulation of fibrillogenesis [151,152]. In previous studies, decorin has been studied in its purified form also as a tumour suppressor [153,154,155,156]. However, a role for decorin in isolated C2C12-EVs has not been investigated. Therefore, in addition to the proteomic analysis, the EV-mediated export of decorin was further confirmed by Western blotting, and by co-staining with CD63. Interestingly, decorin expression in tumours is significantly reduced from the levels expressed in normal tissues [157]. It has been reported that overexpression of decorin could block the cell cycle arrest at G1 phase and inhibit the invasiveness of lung cancer A549 cells causing cell apoptosis and inhibition of metastasis via increased p53 and p21 expression [158]. Decorin overexpression induced apoptosis and cell growth arrest in rat mesangial cells in vitro [40], and roles for decorin in mitophagy and mitochondrial autophagy causing intrinsic apoptosis have also been identified [154,159]. Our observations of C2C12-EV-induced intrinsic apoptosis of CMT 64/61 alongside mitochondrial and lysosomal dysfunction were supported by the raised Bax/Bcl-2 ratio indicating a move toward pro-apoptotic signalling. In addition, decorin negatively regulates insulin-like growth factor receptor I (IGF-IR) [160] and hepatocyte growth factor receptor (Met) [161] and can inhibit tumour cell growth and migration [162]. Decorin is furthermore capable of binding and sequestering growth factors, including TGF-β, in the ECM. In advanced cancer, having acquired resistance to its early growth-inhibitory action, TGF-β becomes pro-oncogenic [163]. This is manifest as EMT induction [164], increased cell proliferation, invasion, metastasis, immune evasion and angiogenesis [164].

Decorin’s multiple interactions with growth factors, their receptors and associated down-stream pathways highlights its multifaceted roles in growth inhibition, metastasis and angiogenesis of tumours [39,165,166,167,168,169]. This is of considerable interest in the context of our current findings, showing export of decorin in myocyte-EVs, which exerted anti-cancer effects on the lung cancer cells, accompanied by effects on migration, growth inhibition, cell cycle arrest, lysosomal changes, mitochondrial damage and apoptosis. In recent years, EVs have indeed been identified as delivery vehicles for myokines [42], and while it is well established that skeletal muscle secretes myokines into the extracellular milieu, reports of skeletal muscle-derived EVs carrying these myokines (including decorin) are scarce. Furthermore, decorin has been reported to be an exercise-regulated myokine which is secreted in response to muscle contractions [170] and associated with major roles in muscle cell differentiation, wound healing, muscular development and regulation of inflammation [156]. Skeletal muscle metastases from lung cancer are rare [171,172]; indeed, in the later stages of cancer with tumour-promoting TGF-β, decorin, which is enriched by skeletal muscle contraction [170], may sequester TGF-β [172]. This would antagonise any tumourigenic properties of TGF-β, ensuring that a pre-metastatic niche is not established [173]. As an abundant cytokine in skeletal muscle, TGF-β is produced by anti-inflammatory macrophages, especially after exercise-induced damage [174,175]. In terms of the therapeutic potential of C2C12-EVs, the anti-tumour potential of decorin could be further tested by overexpressing decorin fused to an EV sorting domain to enable packaging of the overexpressed protein into nascent EVs. To then exploit the clinical translation potential will require approaches summarised elsewhere [176].

EVs released from skeletal muscle have been shown to have cargo signatures that are important for the function of the muscle microenvironment [177]. Interestingly, exercise-induced muscle damage has been shown to modulate EV plasma profiles in different age populations [35] and, therefore, further research into connections between exercise across the life-span and putative anti-cancer effects mediated by C2C12-EV cargoes will be of interest for future studies [178,179]. In cancer-associated cachexia, which leads to loss of skeletal muscle mass, EVs secreted from cancer cells were reported to induce muscle atrophy and modify EV cargoes of C2C12 cells affecting inflammatory, ER stress and mitochondrial pathways [180]. In addition, it may be postulated that sarcopenia, the reduced skeletal muscle mass associated with ageing, may possibly affect pro-cancerous pathways in older age [181,182,183], including via EV-mediated communication. Furthermore, EV-mediated communication linked to changes in skeletal muscle mass may be of interest in relation to health implications caused by space-flight-induced skeletal muscle atrophy, a major health concern in astronauts [184,185,186,187].

In summary, our reported findings suggest that C2C12-EVs from highly viable C2C12 myocytes mediate pathways that suppress carcinoma cells. That the EVs were from myocytes showing high viability implies that the induced apoptosis is not due to apoptosis-inducing molecules from apoptotic myocytes. Murine lung carcinoma cells showed uptake of C2C12-EVs, which suggests subsequent release of EV cargoes. Our inclusion of NIH-3T3 EVs as a functionally inert EV control from a different cell type was essential for interpretation of our EV studies to exclude ‘background’ EV activity and was as we and others have used elsewhere [47,188]. NIH-3T3 EVs, as well as an inert EV control for C2C12-EVs, acted as control for any common EV macromolecules and likely co-isolated proteins that may have had an effect in our experimental system. The use of EV-depleted controls, in which ‘soluble factors’ were separated by SEC from EVs in conditioned medium, was also important [47]. That the non-vesicular, soluble protein isolate only marginally reduced CMT 64/61 viability importantly ruled out the likelihood this effect was due to non-vesicular molecules co-purified with the EVs, and further supported the role of EVs.

Limitations of the current study include the need to test for these inhibitory effects on a variety of cancer cell types. Also, despite the control, soluble factors from the CM (EV-depleted), only marginally reducing cell viability, it is still possible that soluble factors such as adenosine, soluble decorin or other soluble factors released from the ECM (which we found in EVs) may play a role in the observations made. Whilst soluble factors as part of the secretome released from C2C12 myocytes may play a role in inducing apoptosis of the CMT 64/61 cells, this study shows that as a component of that secretome, EVs certainly play a significant role. Where specific EV components are identified, the effects of their soluble equivalents on CMT 64/61 cells, although not within the remit of the current study, could in future also be tested. Future in vitro work will also look at the effect of myocyte EVs on other cancers that induce skeletal muscle metastasis, including melanoma and sarcoma, and these findings will be followed up by in vivo experiments. Our experiments have been in vitro but, even so, we do not suggest that circulating C2C12-EVs would inhibit lung cancers, but would rather suggest that the concentration of C2C12-EVs in situ would inhibit metastasizing cells. Finally, miRNAs within the C2C12-EVs that may activate pro-apoptotic genes or suppress anti-apoptotic genes, as, for example, miR-375 [43] are also being studied.

## 5. Conclusions

Cancer progression in skeletal muscle is very rare, and mechanisms remain unclear. Findings of our current study provide new evidence for anti-cancer roles of skeletal muscle-derived secretome and the EVs therein on lung carcinoma cells, including via lysosomal and mitochondrial pathways. Future investigation on EV-mediated protein/miRNA export in mediating protection against cancer metastasis to skeletal muscle is therefore needed, not just for decorin (identified here in EVs) and previously recognised as a tumour suppressor, but other proteins enriched in C2C12 myocytes involved in lysosomal and mitochondrial pathways, including cathepsins and Bcl-2 proteins. Future work will need to profile the whole range of macromolecules in the C2C12 myocyte secretome. These molecules, such as cytokines, growth factors, lipids and miRNAs, may either be carried within the EVs or be present as soluble factors, free of EVs. They may also comprise part of the EV corona, acquired from the surrounding biofluid, which in in vitro experiments will be a simple, serum-free medium into which the cell has secreted proteins. In the current study, these soluble factors (SF) were themselves shown to have only minimal effects on CMT 64/61 viability. Future studies to compare the relative effects of all these secretome components are essential. These components and their relative contributions to EV- and secretome-mediated effects on cancer cells will also need to be studied in different growth conditions, such as hypoxia, or presence-or-absence of serum, in which EV production and protein cargoes may be affected. Our findings highlight the emerging anti-cancer and likely anti-metastatic roles for C2C12-EVs, and their potential as putative novel therapeutic agents for highly metastatic lung carcinoma.

## Figures and Tables

**Table 1:** List of proteins specific to C2C12-EVs. UniProt acession number, UniProt name, Protein name and Gene symbol are shown.

UniProt Accession	UniProt Name	Protein Name (88/387)	Gene Symbol
A0A0A6YW67	A0A0A6YW67_MOUSE	MCG23377, Predicted pseudogene 8797	*Gm8797*
Q61508	ECM1_MOUSE	Extracellular matrix protein 1	*Ecm1*
P10852	4F2_MOUSE	4F2 cell-surface antigen heavy chain	*Slc3a2*
Q61738	ITA7_MOUSE	Integrin alpha-7	*Itga7*
Q62000	MIME_MOUSE	Mimecan	*Ogn*
A0A0R4J0F8	A0A0R4J0F8_MOUSE	Cartilage intermediate layer protein 1	*Cilp*
P28798	GRN_MOUSE	Progranulin	*Grn*
O88322	NID2_MOUSE	Nidogen-2	*Nid2*
O35639	ANXA3_MOUSE	Annexin A3	*Anxa3 (Anx3)*
Q08879	FBLN1_MOUSE	Fibulin-1	*Fbln1*
P09055	ITB1_MOUSE	Integrin beta-1	*Itgb1*
Q9R118	HTRA1_MOUSE	Serine protease HTRA1	*Htra1*
P31001	DESM_MOUSE	Desmin	*Des*
G3X8T3	G3X8T3_MOUSE	Carboxypeptidase	*Ctsa*
Q64299	CCN3_MOUSE	CCN family member 3	*Ccn3*
P25785	TIMP2_MOUSE	Metalloproteinase inhibitor 2	*Timp2 (Timp-2)*
Q8VDN2	AT1A1_MOUSE	Sodium/potassium-transporting ATPase subunit alpha-1	*Atp1a1*
A0A286YDF5	A0A286YDF5_MOUSE	Myoferlin	*Myof*
Q3TWF3	Q3TWF3_MOUSE	Amyloid-beta A4 protein	*App*
Q03350	TSP2_MOUSE	Thrombospondin-2	*Thbs2 (Tsp2)*
P84078	ARF1_MOUSE	ADP-ribosylation factor 1	*Arf1*
A0A0R4J0I9	A0A0R4J0I9_MOUSE	Prolow-density lipoprotein receptor-related protein 1	*Lrp1*
P97449	AMPN_MOUSE	Aminopeptidase N	*Anpep*
Q8CG14	CS1A_MOUSE	Complement C1s-A subcomponent	*C1sa (C1s)*
E9Q616	E9Q616_MOUSE	AHNAK nucleoprotein	*Ahnak*
Q9WTI7	MYO1C_MOUSE	Unconventional myosin-Ic	*Myo1c*
A0A0A0MQM7	A0A0A0MQM7_MOUSE	Matrilin-2	*Matn2*
P53986	MOT1_MOUSE	Monocarboxylate transporter 1	*Slc16a1*
Q9CPU0	LGUL_MOUSE	Lactoylglutathione lyase	*Glo1*
Q501P1	FBLN7_MOUSE	Fibulin-7	*Fbln7*
A2AUC9	KLH41_MOUSE	Kelch-like protein 41	*Klhl41*
P62821	RAB1A_MOUSE	Ras-related protein	*Rab1A (Rab1)*
Q8BMS2	SPON2_MOUSE	Spondin-2	*Spon2*
A0A1B0GR11_MOUSE	A0A1B0GR11_MOUSE	Transaldolase	*Taldo1*
P55065	PLTP_MOUSE	Phospholipid transfer protein	*Pltp*
Q99P72	RTN4_MOUSE	Reticulon-4	*Rtn4*
P58774	TPM2_MOUSE	Tropomyosin beta chain	*Tpm2*
P33146	CAD15_MOUSE	Cadherin-15	*Cdh15*
Q8R2G6	CCD80_MOUSE	Coiled-coil domain-containing protein 80	*Ccdc80*
A0A0R4J1D0	A0A0R4J1D0_MOUSE	Copine-2	*Cpne2*
O09164	SODE_MOUSE	Extracellular superoxide dismutase	*Sod3*
P35762	CD81_MOUSE	CD81 antigen	*Cd81 (Tapa1)*
G5E829	AT2B1_MOUSE	Plasma membrane calcium-transporting ATPase 1	*Atp2b1*
Q62465	VAT1_MOUSE	Synaptic vesicle membrane protein VAT-1 homologue	*Vat1 (Vat-1)*
G3UXY9	G3UXY9_MOUSE	Ectonucleotide pyrophosphatase	*Enpp2*
Q99K51	PLST_MOUSE	Plastin-3	*Pls3*
Q9WUU7	CATZ_MOUSE	Cathepsin Z	*Ctsz*
Q9D154	ILEUA_MOUSE	Leukocyte elastase inhibitor A	*Serpinb1a*
P12032	TIMP1_MOUSE	Metalloproteinase inhibitor 1	*Timp1*
P39688	FYN_MOUSE	Tyrosine-protein kinase Fyn	*Fyn*
P16110	LEG3_MOUSE	Galectin-3	*Lgals3*
Q6P1B9	Q6P1B9_MOUSE	Bin1 protein	*Bin1*
K3W4Q8	K3W4Q8_MOUSE	Basigin	*Bsg*
P84078/P61205	ARF1_MOUSE/ARF3_MOUSE	ADP-ribosylation factor 1	*Arf1/Arf3*
P97467	AMD_MOUSE	Peptidyl-glycine alpha-amidating monooxygenase	*Pam*
Q9WVJ3	CBPQ_MOUSE	Carboxypeptidase Q	*Cpq*
Q8CG16	C1RA_MOUSE	Complement C1r-A subcomponent	*C1ra (C1r)*
Q62165	DAG1_MOUSE	Dystroglycan	*Dag1 (Dag-1)*
P20060	HEXB_MOUSE	Beta-hexosaminidase subunit beta	*Hexb*
E9Q043	E9Q043_MOUSE	Fibronectin type III domain-containing 1	*Fndc1*
O35887	CALU_MOUSE	Calumenin	*Calu*
Q9D0F9	PGM1_MOUSE	Phosphoglucomutase-1	*Pgm1*
O88325	O88325_MOUSE	Alpha-N-acetylglucosaminidase	*Naglu*
Q5SX40	MYH1_MOUSE	Myosin-1	*Myh1*
P62071	RRAS2_MOUSE	Ras-related protein R-Ras2	*Rras2*
Q07076	ANXA7_MOUSE	Annexin A7	*Anxa7 (Anx7)*
P98064	MASP1_MOUSE	Isoform 2 of Mannan-binding lectin serine protease 1	*Masp1*
P05202	AATM_MOUSE	Aspartate aminotransferase	*Got2 (Got-2)*
O89051	ITM2B_MOUSE	Integral membrane protein 2B	*Itm2b*
Q61187	TS101_MOUSE	Tumour susceptibility gene 101 protein	*Tsg101*
Q6PIE5	AT1A2_MOUSE	Sodium/potassium-transporting ATPase subunit alpha-2	*Atp1a2*
Q8R429	AT2A1_MOUSE	Sarcoplasmic/endoplasmic reticulum calcium ATPase 1	*Atp2a1*
P54116	STOM_MOUSE	Erythrocyte band 7 integral membrane protein	*Stom*
Q9JJH1	RNAS4_MOUSE	Ribonuclease 4	*Rnase4*
O35566	CD151_MOUSE	CD151 antigen	*Cd151*
Q04447	KCRB_MOUSE	Creatine kinase B-type	*Ckb*
O88990	ACTN3_MOUSE	Alpha-actinin-3	*Actn3*
E9Q1X8	E9Q1X8_MOUSE	Voltage-dependent calcium channel subunit alpha-2/delta-1	*Cacna2d1*
Q99JB8	PACN3_MOUSE	Protein kinase C and casein kinase II substrate protein 3	*Pacsin3*
B2RXS4	PLXB2_MOUSE	Plexin-B2	*Plxnb2*
Q8C0E3	TRI47_MOUSE	E3 ubiquitin-protein ligase TRIM47	*Trim47*
Q62351	TFR1_MOUSE	Transferrin receptor protein 1	*Tfrc*
P45591	COF2_MOUSE	Cofilin-2	*Cfl2*
P47880	IBP6_MOUSE	Insulin-like growth factor-binding protein 6	*Igfbp6*
P23927	CRYAB_MOUSE	Alpha-crystallin B chain	*Cryab*
Q80VQ0	AL3B1_MOUSE	Aldehyde dehydrogenase family 3 member B1	*Aldh3b1*

**Table 2:** List of proteins common to both C2C12 myocytes and CMT64/61 carcinoma cells but significantly high in C2C12-EVs. Protein name, accession number and *p*-value are shown.

No.	Protein Name	Accession Number	*p* Value
1	Collagen alpha-1(XII) chain	E9PX70_MOUSE	0.006
2	Isoform 2 of Filamin-C	FLNC_MOUSE	0.036
3	Moesin	MOES_MOUSE	0.021
4	Fibrillin-1	FBN1_MOUSE	0.0032
5	Adipocyte enhancer-binding protein 1	AEBP1_MOUSE	0.013
6	Complement factor H	CFAH_MOUSE	0.0099
7	Prostaglandin F2 receptor negative regulator	FPRP_MOUSE	0.023
8	Biglycan	PGS1_MOUSE	0.035
9	Cathepsin B	CATB_MOUSE	0.0092
10	SPARC	A0A1L1SSH9_MOUSE	0.011
11	Cathepsin L1	CATL1_MOUSE	0.041
12	Prosaposin	E9PZ00_MOUSE	0.022
13	Endoplasmic reticulum chaperone BiP	BIP_MOUSE	0.0096
14	Serine (or cysteine) peptidase inhibitor	F8WIV2_MOUSE	0.02
15	EH domain-containing protein 1	EHD1_MOUSE	0.044
16	Sulfhydryl oxidase 1	QSOX1_MOUSE	0.015
17	Neural cell adhesion molecule 1	A0A0A6YY47_MOUSE	0.0068
18	EH domain-containing protein 4	EHD4_MOUSE	0.04
19	Decorin	PGS2_MOUSE	0.028

## Data Availability

The data is contained within the article and Supplementary Materials.

## Supplementary Materials

The following supporting information can be downloaded at: https://www.mdpi.com/article/10.3390/biology14111578/s1, Figures S1–S5 and Table S1. Figure S1 Differentiation of C2C12 myoblasts to myocytes and Myogenin expression of differentiated C2C12 myocytes. (A), Bright field image of C2C12 myoblast cells prior to differentiation; scale bar: 100 µm. (B), Bright field image of differentiated C2C12 (myocytes), at day 4. White arrows show fusion and formation of syncytia; scale bar: 200 µm. (C), a 30 kDa band identified as Myogenin in western blot analysis. Lane 1: C2C12 myoblasts treated with growth medium (GM) for 24 h. Lane 2: C2C12 cells treated with differentiation medium (DM) for 24 h. Lane 3: C2C12 cells treated with differentiation medium for 4 days. To compare myogenin intensities at days 1 and 4 of differentiation, band intensities were quantified and normalised to β-actin (43 kDa) (0.190 day 1; 0.667, day 2) using Image J 1.52p and fold-changes calculated (bar graph). Figure S2 Characterisation of C2C12 EVs by NTA, DLS, TEM and protein marker. (A), Representative NTA histogram showing EV size and concentration. NTA of this sample shows 3 populations of C2C12 EVs with modal peak diameters of 138 nm, 228 nm and 337 nm. (B), Average data on C2C12 EV size detected by NTA, showing peak modal size of 137.7 ± 5.6 nm and D10, D50 and D90 indicating the point in the size distribution in which 10, 50 and 90% of the sample is contained, respectively. (C), Size Distribution by number using DLS. (D), DLS determination of Zeta Potential of C2C12 EVs (n = 7). (E), TEM of EVs isolated from C2C12 murine myocytes showing characteristic spheroid morphology; range of diameters: 100–150 nm; scale 100 nm. (F), Representative western analysis (n = 3) showing expression of TSG101 (cytosolic protein) and CD63 (transmembrane protein) on C2C12 EVs but not the EV contaminant negative marker, GM130. (G), Fractions recovered from SEC of CM from C2C12 myocytes were analysed for total protein (Ponceau S staining) and for EV marker CD81 by western blot. Figure S3 Effect of serum concentration on growth of CMT 64/61. (A), CMT 64/61 cells were grown with or without a range of serum concentrations over 5 days. An increased growth rate of CMT 64/61 in 5 and 10% serum was observed for cells grown in Waymouth’s MB 752/1 medium (WM). The growth rate of cells maintained in 5% EV-depleted serum-free conditions was significantly lower compared to cells in 5% serum only by day 4 (*p* = 0.023). Up to 48 h, however, there was no significance difference (p = 0.143). Growth of CMT 64/61 in 2% EV-depleted FBS in DMEM at the 48, 72 and 96 h points was not significantly below that found in 2% EV-depleted FBS in WM (green squares) (*p* = 0.1 (48 h), *p* = 0.15 (72 h), *p* = 0.11 (96 h)). (B), NIH-3T3 cells or (C), C2C12 cells grown in DMEM with 2% FBS or 2% FBS (EV-depleted). Figure S4 Metastatic murine lung carcinoma cells (CMT 64/61) fail to invade/colonise C2C12 skeletal muscle cells but do invade/colonise fibroblast (NIH-3T3) control cells and C2C12 conditioned medium inhibits proliferation of CMT 64/61 lung carcinoma cells. (A), represents a normal morphology of differentiated skeletal muscle cells (C2C12). Carcinoma cells (CMT 64/61) were co-cultured with skeletal muscle cells (C2C12) at a low ratio (CMT 64/61:C2C12; 0.025:1), (B) and at a higher ratio (0.25:1), (C); there was no sign of colonisation of the C2C12 cells by the CMT 64/61 cells. (D), demonstrates a normal morphology of NIH-3T3 after 48 h. In (E), carcinoma cells (CMT 64/61) were co-cultured with fibroblasts at a 0.025:1 ratio, showing cellular dysplasia of the fibroblast cells and in (F) where carcinoma cells were co-cultured with fibroblasts at a higher ratio of 0.25:1 signs of invasion and colonisation of the fibroblast cells by the carcinoma cells was visible (area delineated by yellow dotted line showing invading carcinoma cells). (G), CMT 64/61 grown under normal conditions. Scale bar, 100 μm. Cell viability analysis of CMT 64/61 carcinoma cells treated with C2C12 and NIH-3T3 conditioned medium (CM), (H). CM at a range of concentrations was added to CMT 64/61 cells (48 h/37 °C), cells were stained with Guava ViaCount reagent and analysed by flow cytometry. The data presents the mean ± standard deviation of one of two experiments performed in triplicate; * *p* < 0.05 was considered statistically significant. Photomicrographs of CMT 64/61 cells treated with increasing protein concentrations of C2C12 and NIH 3T3 CM (48 h; 37 °C) (I); scale bar = 100 μm. Figure S5 Analysis of PKH26 labelled myocyte EVs C2C12 EVs were isolated by centrifugation and labelled with PKH26 dye according to the manufacturer’s protocol. (A), Fluorescence microscopy of PKH26-labelled EVs in liquid medium showing aggregation of vesicles as indicated by white arrows. Scale bar= 400 µm. (B), Fluorescence-NTA showing the PKH26-labelled C2C12 EVs (approximately 62% showing PKH fluorescence). (C), NTA profiles of unlabelled C2C12 EVs and PKH26-labelled EVs. (D), Standard curve of fluorescence intensity versus concentration of EV protein. Values represent mean ± SD from three independent experiments. Fluorescence intensity showed a strong linear relationship with EV protein concentration (inset), ranging from 0–50 μg/ml. Table S1: Table of Proteins showing proteins present or absent in C2C12 myocytes and NIH-3T3 Extracellular Vesicles identified by liquid chromatography with tandem mass spectrometry (LC-MS/MS) analysis.
