# Supplementary material for: Extracellular Vesicles from the Myocyte Secretome Contribute In Vitro to Creating an Unfavourable Environment for Migrating Lung Carcinoma Cells
Source: Biology (Basel). 2025 Nov 11;14(11):1578. doi: 10.3390/biology14111578 (PMC12650371; doi:10.3390/biology14111578)
Supplement: Supplementary file 1 [file biology-14-01578-s001.zip › Table S1.pdf]

Table S1: Table of Proteins showing proteins present or absent in C2C12 myocytes and NIH-3T3 Extracellular Vesicles identified by liquid chromatography with tandem mass spectrometry (LC-MS/MS) analysis

Proteins present assigned (+), proteins absent assigned (-).

No.	Protein Name	Myocyte EVs	NIH-3T3 EVs
1	14-3-3 protein epsilon	+	+
2	14-3-3 protein gamma	+	+
3	14-3-3 protein theta (Fragment)	-	+
4	14-3-3 protein zeta/delta	+	+
5	26S proteasome non-ATPase regulatory subunit 2	-	+
6	26S proteasome non-ATPase regulatory subunit 6	-	+
7	40S ribosomal protein S11	-	+
8	40S ribosomal protein S16	-	+
9	40S ribosomal protein S3	-	+
10	40S ribosomal protein S3a	-	+
11	40S ribosomal protein S4, X isoform	-	+
12	40S ribosomal protein S7	-	+
13	40S ribosomal protein S9	-	+
14	40S ribosomal protein SA	-	+
15	60S acidic ribosomal protein P0	-	+
16	60S ribosomal protein L12	-	+
17	6-phosphogluconate dehydrogenase, decarboxylating	-	+
18	72 kDa type IV collagenase	+	+
19	Acetyl-CoA acetyltransferase, cytosolic	-	+
20	Actin, alpha skeletal muscle	+	+
21	Actin, cytoplasmic 1	+	+
22	Actin-related protein 2	-	+
23	Actin-related protein 2/3 complex subunit 4	-	+
24	Actin-related protein 3	-	+
25	Adenosylhomocysteinase	-	+
26	Adenylosuccinate synthetase isozyme 2	-	+
27	Adipocyte enhancer-binding protein 1	+	+
28	ADP-ribosylation factor 1	+	-
29	AHNAK nucleoprotein (desmoyokin)	+	-
30	Alanine--tRNA ligase, cytoplasmic	-	+
31	Alcohol dehydrogenase [NADP(+)]	-	+
32	Aldehyde dehydrogenase family 3 member B1	+	-
33	Aldose reductase	+	+
34	Alpha-actinin-1	+	+
35	Alpha-actinin-3	+	-
36	Alpha-actinin-4	+	+
37	Alpha-crystallin B chain	+	-
38	Alpha-enolase	+	+
39	Alpha-N-acetylglucosaminidase	+	-
40	Aminopeptidase N	+	-
41	Amyloid-beta A4 protein	+	-
42	Annexin A1	+	+
43	Annexin A2	+	+
44	Annexin A3	+	-
45	Annexin A5	+	+
46	Annexin A6	+	+

47	Annexin A7	+	-
48	Arginine--tRNA ligase, cytoplasmic	-	+
49	Asparagine synthetase [glutamine- hydrolyzing]	-	+
50	Asparagine--tRNA ligase, cytoplasmic	-	+
51	Aspartate aminotransferase, cytoplasmic	+	+
52	Aspartate aminotransferase, mitochondrial	+	-
53	Aspartate--tRNA ligase, cytoplasmic	-	+
54	ATP-citrate synthase	-	+
55	ATP-dependent RNA helicase A	-	+
56	Basement membrane-specific heparan sulfate proteoglycan core protein	+	+
57	Basigin	+	-
58	Beta-enolase	+	+
59	Beta-hexosaminidase subunit beta	+	-
60	Bifunctional glutamate/proline--tRNA ligase	-	+
61	Bifunctional purine biosynthesis protein PURH	-	+
62	Biglycan	+	+
63	Bin1 protein	+	-
64	Bone morphogenetic protein 1	+	+
65	Cadherin-15	+	-
66	Calpain-6	-	+
67	Calreticulin	+	+
68	Calsequestrin-2	+	+
69	Calsyntenin-1	+	+
70	Calumenin	+	-
71	Carboxypeptidase	+	-
72	Carboxypeptidase Q	+	-
73	Cartilage intermediate layer protein 1	+	-
74	Cathepsin B	+	+

75	Cathepsin D	+	+
76	Cathepsin L1	+	+
77	Cathepsin Z	+	-
78	CD151 antigen	+	-
79	CD81 antigen	+	-
80	CD82 antigen	+	+
81	Ceruloplasmin	-	+
82	Chloride intracellular channel protein 1	-	+
83	Clathrin heavy chain 1	+	+
84	Coatomer subunit alpha	-	+
85	Coatomer subunit beta	-	+
86	Coatomer subunit beta'	-	+
87	Cofilin-1	+	+
88	Cofilin-2	+	-
89	Coiled-coil domain-containing protein 80	+	-
90	Collagen alpha-1(I) chain	+	+
91	Collagen alpha-1(III) chain	+	+
92	Collagen alpha-1(V) chain	+	+
93	Collagen alpha-1(VI) chain	+	+
94	Collagen alpha-1(XII) chain	+	+
95	Collagen alpha-2(I) chain	+	+
96	Collagen alpha-2(IV) chain	-	+
97	Collagen alpha-2(V) chain	+	+
98	Collagen alpha-2(VI) chain	+	+
99	Collagen, type VI, alpha 3	+	+
100	Complement C1r-A subcomponent	+	-
101	Complement C1s-A subcomponent	+	-
102	Complement factor H	+	+
103	Copine-2	+	-

104	Creatine kinase B-type	+	-
105	Cullin-associated NEDD8-dissociated protein 1	-	+
106	Cystatin-C	+	+
107	Cytoplasmic dynein 1 heavy chain 1	-	+
108	Cytoplasmic dynein 1 intermediate chain 2	-	+
109	D-3-phosphoglycerate dehydrogenase	-	+
110	DEAD (Asp-Glu-Ala-Asp) box polypeptide 17, isoform CRA	-	+
111	Decorin	+	+
112	Desmin	+	-
113	Desmoplakin	+	+
114	Dihydropyrimidinase-related protein 2	-	+
115	DNA damage-binding protein 1	-	+
116	DNA replication licensing factor MCM2	-	+
117	DNA-(apurinic or apyrimidinic site) lyase	-	+
118	Dystroglycan	+	-
119	Ectonucleotide pyrophosphatase	+	-
120	EGF-containing fibulin-like extracellular matrix protein 2	-	+
121	EH domain-containing protein 1	+	+
122	EH domain-containing protein 4	+	+
123	Elongation factor 1-alpha 1	+	+
124	Elongation factor 1-delta	-	+
125	Elongation factor 1-gamma	-	+
126	Elongation factor 2	+	+
127	EMILIN-1	-	+
128	Endoplasmic reticulum chaperone BiP	+	+
129	Endoplasmin	+	+
130	Erythrocyte band 7 integral membrane protein	+	-
131	Eukaryotic initiation factor 4A-I	+	+

132	Eukaryotic peptide chain release factor subunit 1	-	+
133	Eukaryotic translation initiation factor 2 subunit 1	-	+
134	Eukaryotic translation initiation factor 2 subunit 3, X-linked	-	+
135	Eukaryotic translation initiation factor 3 subunit A	-	+
136	Eukaryotic translation initiation factor 3 subunit C	-	+
137	Eukaryotic translation initiation factor 3 subunit E	-	+
138	Eukaryotic translation initiation factor 3 subunit I	-	+
139	Eukaryotic translation initiation factor 3 subunit L	-	+
140	Eukaryotic translation initiation factor 5A (Fragment)	-	+
141	Exportin-1	-	+
142	Exportin-2	-	+
143	Extracellular matrix protein 1	+	-
144	Extracellular superoxide dismutase [Cu-Zn]	+	-
145	F-actin-capping protein subunit alpha-1	-	+
146	Farnesyl pyrophosphate synthase	-	+
147	Fascin	-	+
148	Fatty acid synthase	-	+
149	Fatty acid-binding protein, epidermal	-	+
150	Fibrillin-1	+	+
151	Fibronectin	+	+
152	Fibronectin type III domain-containing 1	+	-
153	Fibulin-1	+	-
154	Fibulin-7	+	-
155	Filamin, alpha	-	+
156	Follistatin-related protein 1	+	+
157	Fructose-bisphosphate aldolase A	+	+
158	Galectin-1	+	+
159	Galectin-3	+	-
160	Galectin-3-binding protein	+	+

161	Glucose-6-phosphate isomerase	+	+
162	Glutaredoxin-3	-	+
163	Glutathione S-transferase P 1	-	+
164	Glyceraldehyde-3-phosphate dehydrogenase	+	+
165	Glycine--tRNA ligase	-	+
166	Glypican-1	+	+
167	GMP synthase [glutamine-hydrolyzing]	-	+
168	Granulins	+	-
169	GTP-binding nuclear protein Ran	-	+
170	Guanine nucleotide-binding protein G(i) subunit alpha-2	+	+
171	Guanine nucleotide-binding protein G(I)/G(S)/G(T) subunit beta-2	+	+
172	Heat shock 70 kDa protein 4	+	+
173	Heat shock cognate 71 kDa protein	+	+
174	Heat shock protein HSP 90-alpha	+	+
175	Heat shock protein HSP 90-beta	+	+
176	Hepatoma-derived growth factor	-	+
177	Heterogeneous nuclear ribonucleoprotein A/B	-	+
178	Heterogeneous nuclear ribonucleoprotein A1	-	+
179	Heterogeneous nuclear ribonucleoprotein A3	-	+
180	Heterogeneous nuclear ribonucleoprotein K (Fragment)	-	+
181	Heterogeneous nuclear ribonucleoprotein L (Fragment)	-	+
182	Heterogeneous nuclear ribonucleoprotein R	-	+
183	Heterogeneous nuclear ribonucleoproteins A2/B1	+	+
184	High mobility group protein B1	-	+
185	Histone H1.2	-	+
186	Histone H2B type 1-B	-	+
187	Histone H4	+	+
188	Histone-binding protein RBBP7	-	+
189	Hsc70-interacting protein	+	+
190	Immunoglobulin superfamily member 8	+	+
191	Importin subunit beta-1	-	+
192	Importin-7	-	+
193	Insulin-like growth factor-binding protein 5	+	+
194	Insulin-like growth factor-binding protein 6	+	-
195	Integral membrane protein 2B	+	-
196	Integrin beta-1	+	-
197	Inter-alpha trypsin inhibitor, heavy chain 2	-	+
198	Isocitrate dehydrogenase [NADP] cytoplasmic	-	+
199	Isoform 2 of 4F2 cell-surface antigen heavy chain	+	-
200	Isoform 2 of Choline transporter-like protein 2	+	-
201	Isoform 2 of Fibulin-2	+	+
202	Isoform 2 of Filamin-C	+	+
203	Isoform 2 of Gelsolin	+	+
204	Isoform 2 of Heterogeneous nuclear ribonucleoprotein U	-	+
205	Isoform 2 of Lactadherin	+	+
206	Isoform 2 of Mannan-binding lectin serine protease 1	+	-
207	Isoform 2 of Nestin	+	-
208	Isoform 2 of Periostin	+	+
209	Isoform 2 of Spectrin alpha chain, non-erythrocytic 1	-	+
210	Isoform 2 of Sulfhydryl oxidase 1	+	+
211	Isoform 2 of Tenascin	+	+
212	Isoform 2 of Transcription intermediary factor 1-beta	-	+
213	Isoform 2 of Tripartite motif-containing protein 47	+	-
214	Isoform 2 of Tropomyosin alpha-3 chain	-	+
215	Isoform 2 of Unconventional myosin-Ic	+	-
216	Isoform 2 of V-type proton ATPase catalytic subunit A	-	+
217	Isoform 3 of Heterogeneous nuclear ribonucleoprotein D0	-	+

218	Isoform Alpha-7X2A of Integrin alpha-7	+	-
219	Isoform C1 of Heterogeneous nuclear ribonucleoproteins C1/C2	-	+
220	Isoform PLEC-1D of Plectin	+	+
221	Isoleucine--tRNA ligase, cytoplasmic	-	+
222	Junction plakoglobin	+	+
223	Kelch-like protein 41	+	-
224	Keratin, type I cytoskeletal 14	+	+
225	Keratin, type I cytoskeletal 16	+	+
226	Keratin, type I cytoskeletal 17	+	+
227	Keratin, type II cytoskeletal 5	+	+
228	Keratin, type II cytoskeletal 6A	+	+
229	Lactoylglutathione lyase	+	-
230	Laminin subunit beta-1	-	+
231	Laminin subunit gamma-1	-	+
232	Leucine--tRNA ligase, cytoplasmic	-	+
233	Leukocyte elastase inhibitor A	+	-
234	Leukotriene A-4 hydrolase	-	+
235	L-lactate dehydrogenase	+	+
236	Low density lipoprotein receptor-related protein 1	+	-
237	Lysine--tRNA ligase	-	+
238	Lysyl oxidase homolog 1	+	+
239	Major vault protein	+	+
240	Malate dehydrogenase, cytoplasmic	+	+
241	Malate dehydrogenase, mitochondrial	+	+
242	Matrilin-2	+	-
243	MCG116562, isoform CRA_a	+	+
244	MCG116671	-	+
245	MCG23377, isoform CRA_b	+	-

246	Mesothelin	+	+
247	Metalloproteinase inhibitor 1	+	-
248	Metalloproteinase inhibitor 2	+	-
249	Mimecan	+	-
250	Mitotic checkpoint protein BUB3	-	+
251	Moesin	+	+
252	Monocarboxylate transporter 1	+	-
253	Multifunctional protein ADE2	-	+
254	Myoferlin	+	-
255	Myosin-1	+	-
256	Myosin-3	+	+
257	Myosin-9	+	+
258	Neural cell adhesion molecule 1	+	+
259	Neutral alpha-glucosidase AB	-	+
260	Nidogen-1	-	+
261	Nidogen-2	+	-
262	Non-POU domain-containing octamer-binding protein	-	+
263	Nuclease-sensitive element-binding protein 1	-	+
264	Nucleobindin-1	+	+
265	Nucleolin	+	+
266	Nucleophosmin	-	+
267	Nucleoside diphosphate kinase	+	+
268	Olfactomedin-like protein 3	+	+
269	Peptidyl-glycine alpha-amidating monooxygenase	+	-
270	Peptidyl-prolyl cis-trans isomerase A	+	+
271	Peptidyl-prolyl cis-trans isomerase B	-	+
272	Peptidyl-prolyl cis-trans isomerase FKBP4	-	+
273	Peroxidasin homolog	+	+
274	Peroxiredoxin-1	+	+

275	Peroxiredoxin-2	-	+
276	Phenylalanine--tRNA ligase alpha subunit	-	+
277	Phenylalanine--tRNA ligase beta subunit	-	+
278	Phosphatidylethanolamine-binding protein 1	-	+
279	Phosphoglucomutase-1	+	-
280	Phosphoglycerate kinase 1	+	+
281	Phosphoglycerate mutase 1	+	+
282	Phospholipid transfer protein	+	-
283	Phosphoserine aminotransferase	+	+
284	Pigment epithelium-derived factor	+	+
285	Plasma membrane calcium-transporting ATPase 1	+	-
286	Plastin-3 (Fragment)	+	-
287	Platelet-activating factor acetylhydrolase IB subunit alpha	-	+
288	Platelet-activating factor acetylhydrolase	-	+
289	Plexin-B2	+	-
290	Poly(rC)-binding protein 1	-	+
291	Polyadenylate-binding protein 1	-	+
292	Predicted pseudogene 5580	-	+
293	Prelamin-A/C	+	+
294	Pre-mRNA-processing factor 19	-	+
295	Pre-mRNA-splicing factor ATP-dependent RNA helicase DHX15	-	+
296	Probable ATP-dependent RNA helicase DDX5	-	+
297	Probable ATP-dependent RNA helicase DDX6	-	+
298	Procollagen C-endopeptidase enhancer 1	+	+
299	Procollagen-lysine,2-oxoglutarate 5-dioxygenase 1	+	+
300	Procollagen-lysine,2-oxoglutarate 5-dioxygenase 3	-	+
301	Profilin-1	+	+
302	Programmed cell death 6-interacting protein	+	+

303	Proliferating cell nuclear antigen	-	+
304	Proliferation-associated 2G4	-	+
305	Prolyl endopeptidase	+	+
306	Prosaposin	+	+
307	Prostaglandin F2 receptor negative regulator	+	+
308	Proteasome subunit alpha type-3	-	+
309	Proteasome subunit alpha type-6	-	+
310	Proteasome subunit alpha type-7	+	+
311	Proteasome subunit beta type-5	+	+
312	Protein arginine N-methyltransferase 1	-	+
313	Protein disulfide-isomerase A3	+	+
314	Protein disulfide-isomerase	+	+
315	Protein kinase C and casein kinase II substrate protein 3	+	-
316	Protein kinase C and casein kinase substrate in neurons protein 2	+	-
317	Protein NOV homolog	+	-
318	Protein RCC2	-	+
319	Protein SET (Fragment)	-	+
320	Puromycin-sensitive aminopeptidase	-	+
321	Pyruvate kinase PKM	+	+
322	Rab GDP dissociation inhibitor beta	+	+
323	RAB1A, member RAS oncogene family	+	-
324	RAS-related C3 botulinum substrate 1, isoform CRA_a	-	+
325	Ras-related protein Rab-7a	+	+
326	Ras-related protein R-Ras2	+	-
327	Receptor of activated protein C kinase 1	-	+
328	Reticulon-4	+	-
329	Rho GDP-dissociation inhibitor 1	+	+
330	Ribonuclease 4	+	-

331	Ribonuclease inhibitor	-	+
332	Ribosomal protein	-	+
333	RuvB-like 1	-	+
334	Sarcoplasmic/endoplasmic reticulum calcium ATPase 1	+	-
335	Septin-11	-	+
336	Septin-2	-	+
337	Septin-7	-	+
338	Serine (or cysteine) peptidase inhibitor, clade B, member 6a	+	+
339	Serine protease HTRA1	+	-
340	Serine/threonine-protein phosphatase 2A 65 kDa regulatory subunit A alpha isoform	-	+
341	Serine/threonine-protein phosphatase 2A catalytic subunit alpha isoform	-	+
342	Serine/threonine-protein phosphatase PP1-alpha catalytic subunit	-	+
343	Serine-threonine kinase receptor-associated protein	-	+
344	Serine--tRNA ligase, cytoplasmic	-	+
345	Serpin H1	-	+
346	Sodium/potassium-transporting ATPase subunit alpha-1	+	-
347	Sodium/potassium-transporting ATPase subunit alpha-2	+	-
348	SPARC	+	+
349	Spectrin beta chain, non-erythrocytic 1	-	+
350	Spliceosome RNA helicase Ddx39b	-	+
351	Splicing factor 3B subunit 1	-	+
352	Spondin-2	+	-
353	Staphylococcal nuclease domain-containing protein 1	-	+
354	Stress-induced-phosphoprotein 1	-	+
355	SUMO-activating enzyme subunit 2	-	+
356	Synaptic vesicle membrane protein VAT-1 homolog	+	-
357	Talin-1	+	+
358	T-complex protein 1 subunit alpha	-	+
359	T-complex protein 1 subunit beta	-	+
360	T-complex protein 1 subunit delta	-	+
361	T-complex protein 1 subunit epsilon	-	+
362	T-complex protein 1 subunit eta	-	+
363	T-complex protein 1 subunit gamma	-	+
364	T-complex protein 1 subunit theta	-	+
365	T-complex protein 1 subunit zeta	-	+
366	Thrombospondin-1	+	+
367	Thrombospondin-2	+	-
368	Transaldolase	+	-
369	Transferrin receptor protein 1	+	-
370	Transgelin-2	-	+
371	Transitional endoplasmic reticulum ATPase	+	+
372	Transketolase	-	+
373	Translationally-controlled tumor protein	+	+
374	Triosephosphate isomerase	+	+
375	Tropomyosin beta chain	+	-
376	Tubulin alpha-1A chain	+	+
377	Tubulin beta-5 chain	+	+
378	Tubulin beta-6 chain	-	+
379	Tumor susceptibility gene 101 protein	+	-
380	Tyrosine-protein kinase Fyn	+	-
381	Ubiquitin-like modifier-activating enzyme 1	-	+
382	Vacuolar protein sorting-associated protein 35	-	+

383	Valine--tRNA ligase	-	+
384	Vimentin	+	+
385	Vinculin	+	+
386	Voltage-dependent calcium channel subunit alpha-2/delta-1	+	-
387	WD repeat-containing protein 1	-	+
