# Supplementary material for: Extracellular Vesicles from the Myocyte Secretome Contribute In Vitro to Creating an Unfavourable Environment for Migrating Lung Carcinoma Cells
Source: Biology (Basel). 2025 Nov 11;14(11):1578. doi: 10.3390/biology14111578 (PMC12650371; doi:10.3390/biology14111578)
Supplement: Supplementary file 1 [file biology-14-01578-s001.zip › biology-3905915-WB.pdf]

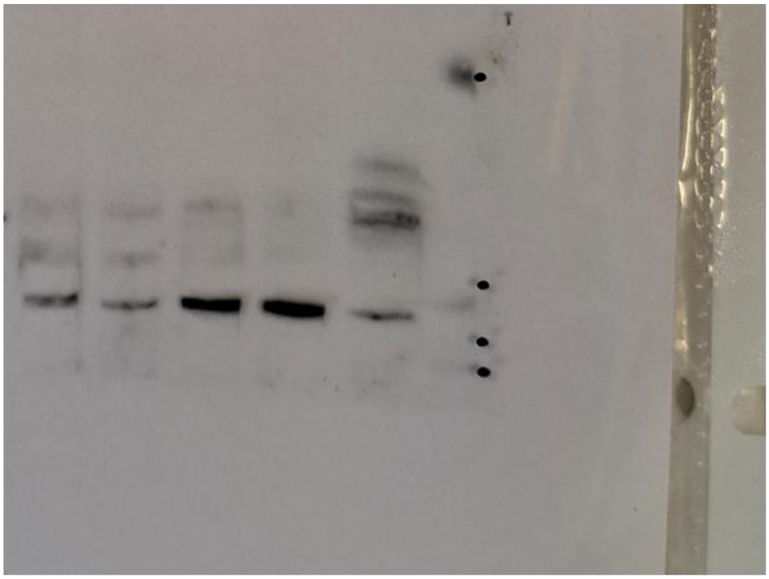

Bax

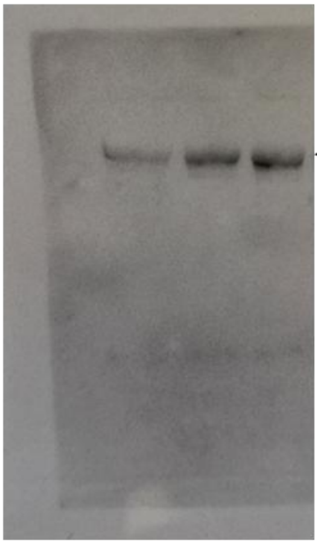

Bcl-2

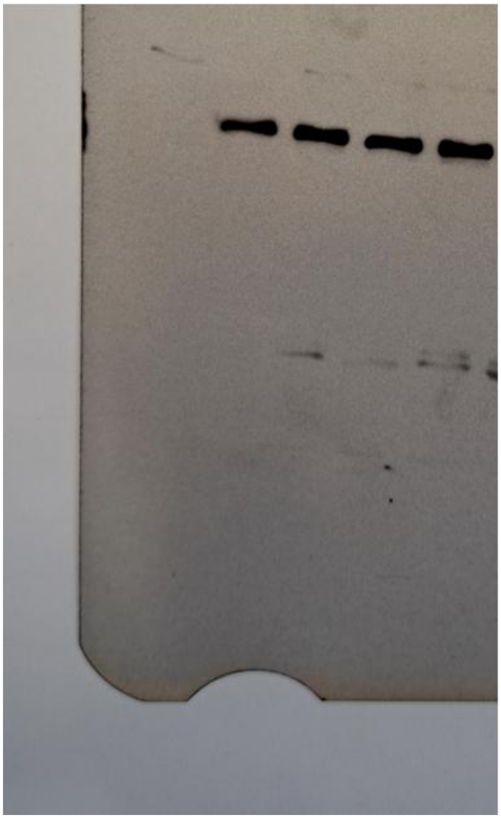

β-actin

Supplementary Figure S2D

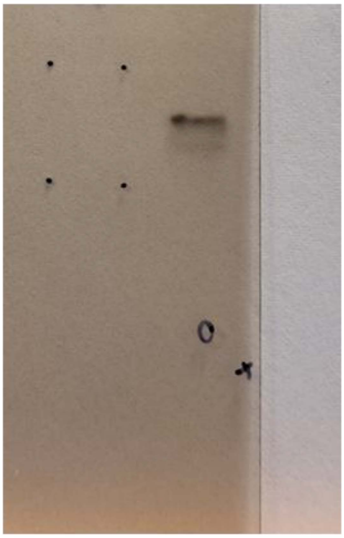

GM130

Supplementary Figure S2G

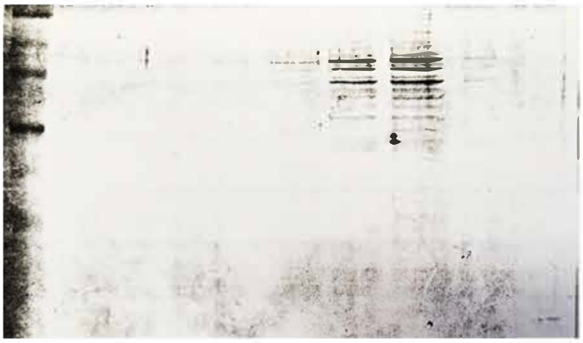

Ponceau S

Supplementary Figure S2E

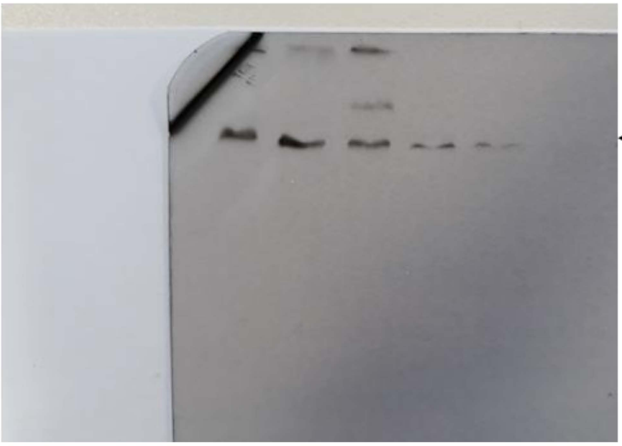

CD81
