# Supplementary figures and images for: Extracellular Vesicles from the Myocyte Secretome Contribute In Vitro to Creating an Unfavourable Environment for Migrating Lung Carcinoma Cells
Source: Biology (Basel). 2025 Nov 11;14(11):1578. doi: 10.3390/biology14111578 (PMC12650371; doi:10.3390/biology14111578)

Figure S1

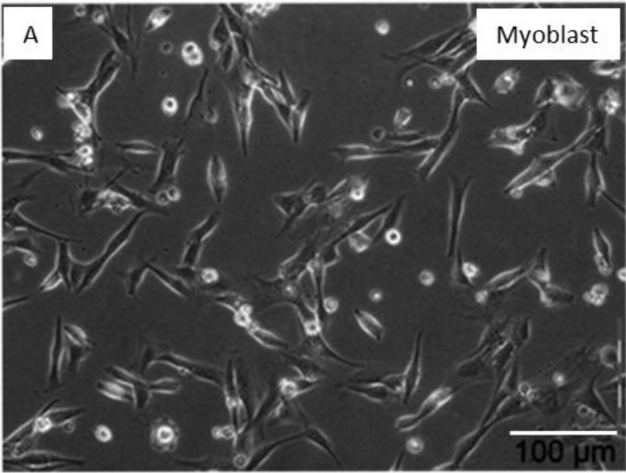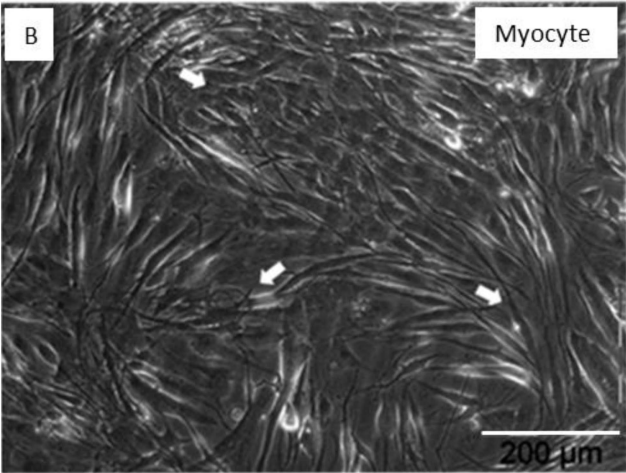

**C**

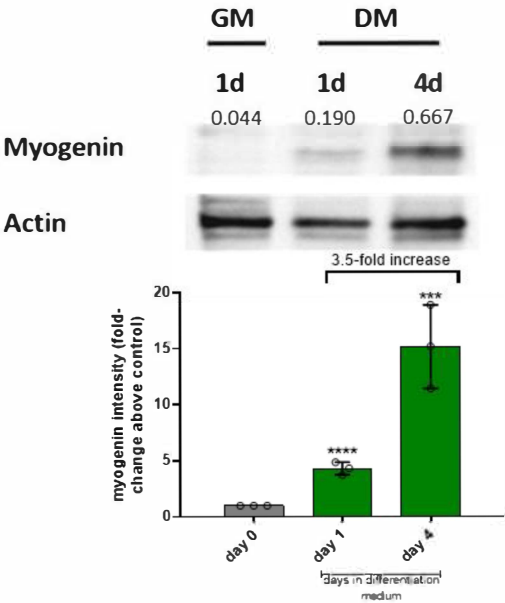

Figure S2

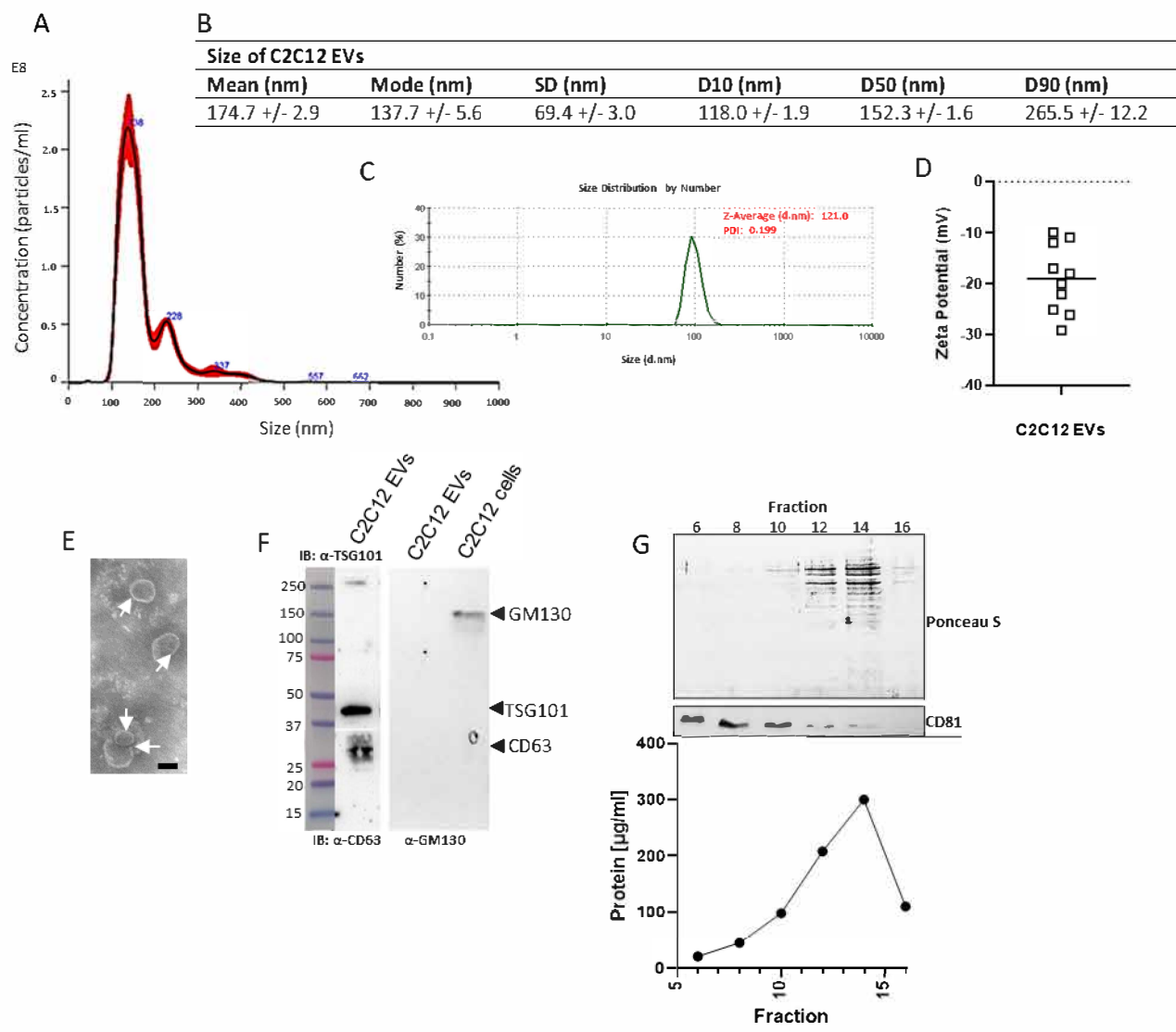

Figure S3

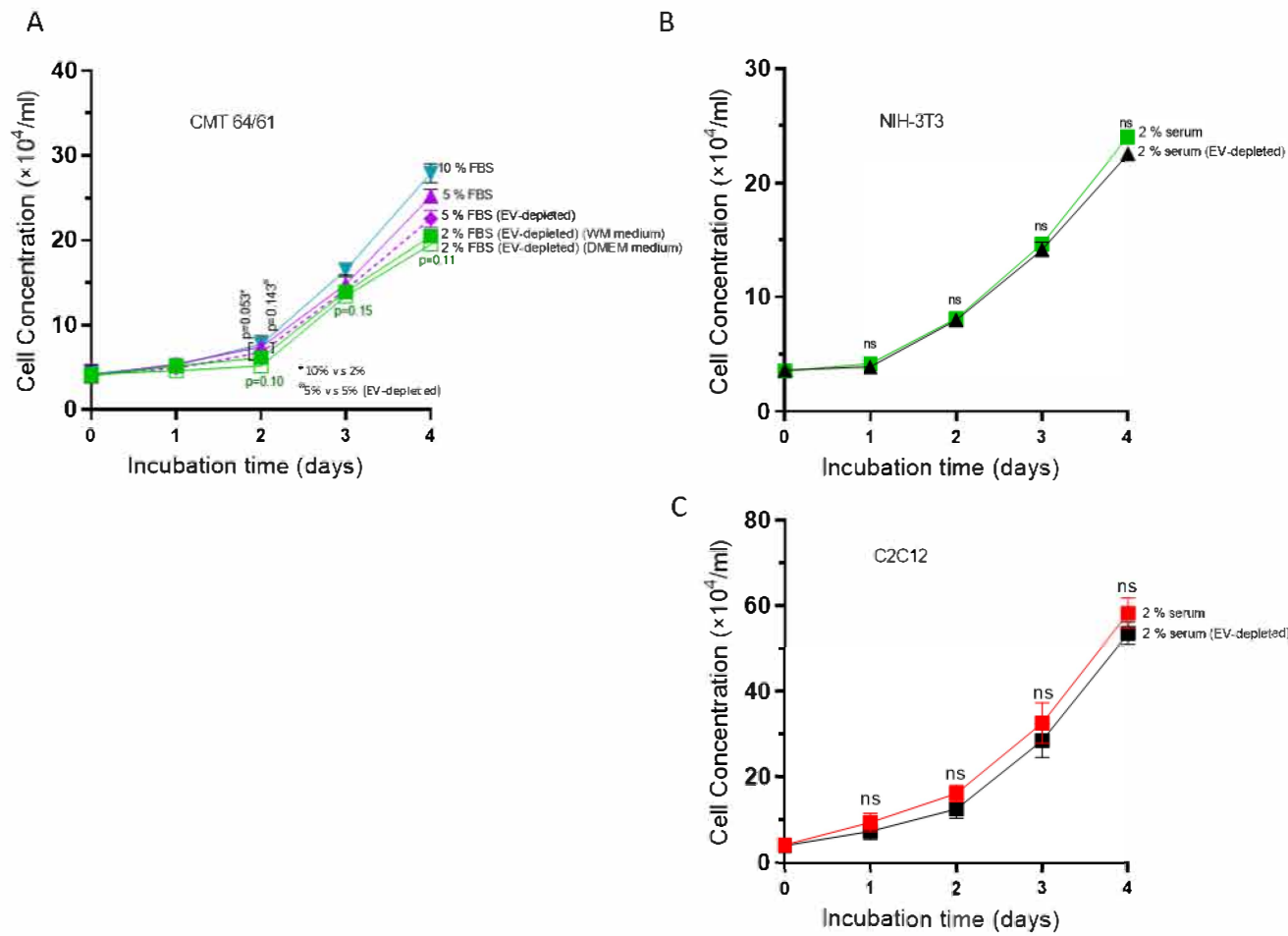

Figure S4

CMT 64/61 : C2C12

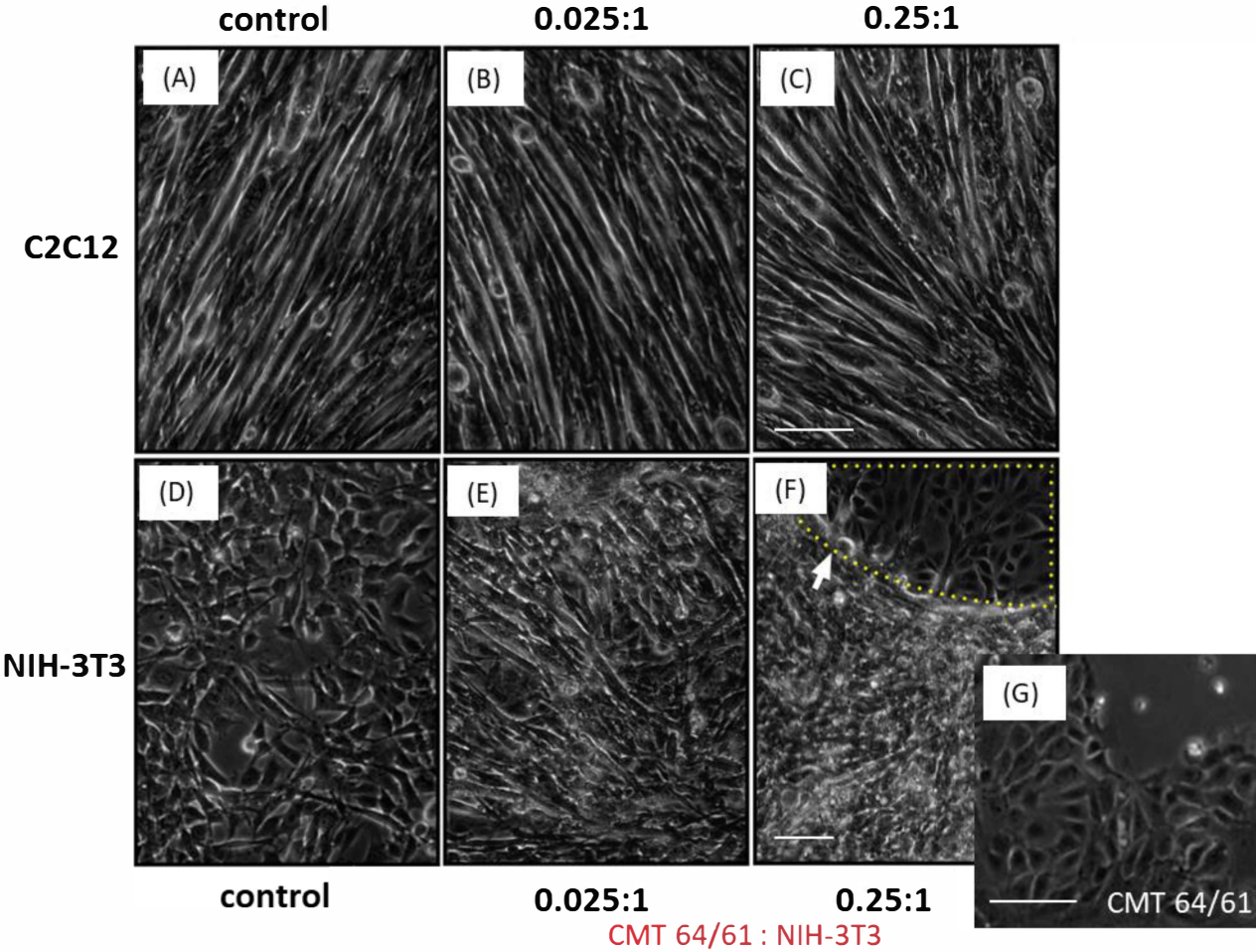

(H)

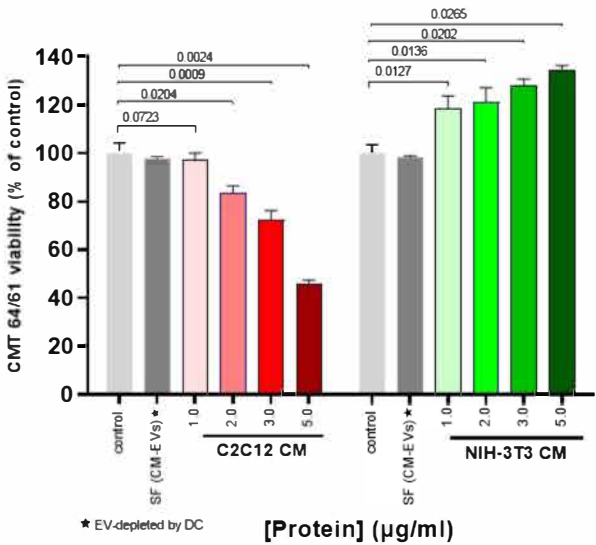

(I)

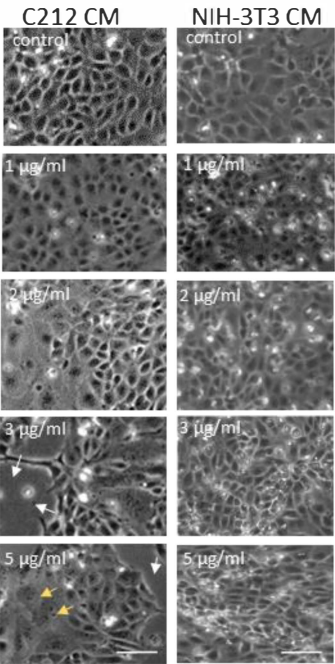

Figure S5

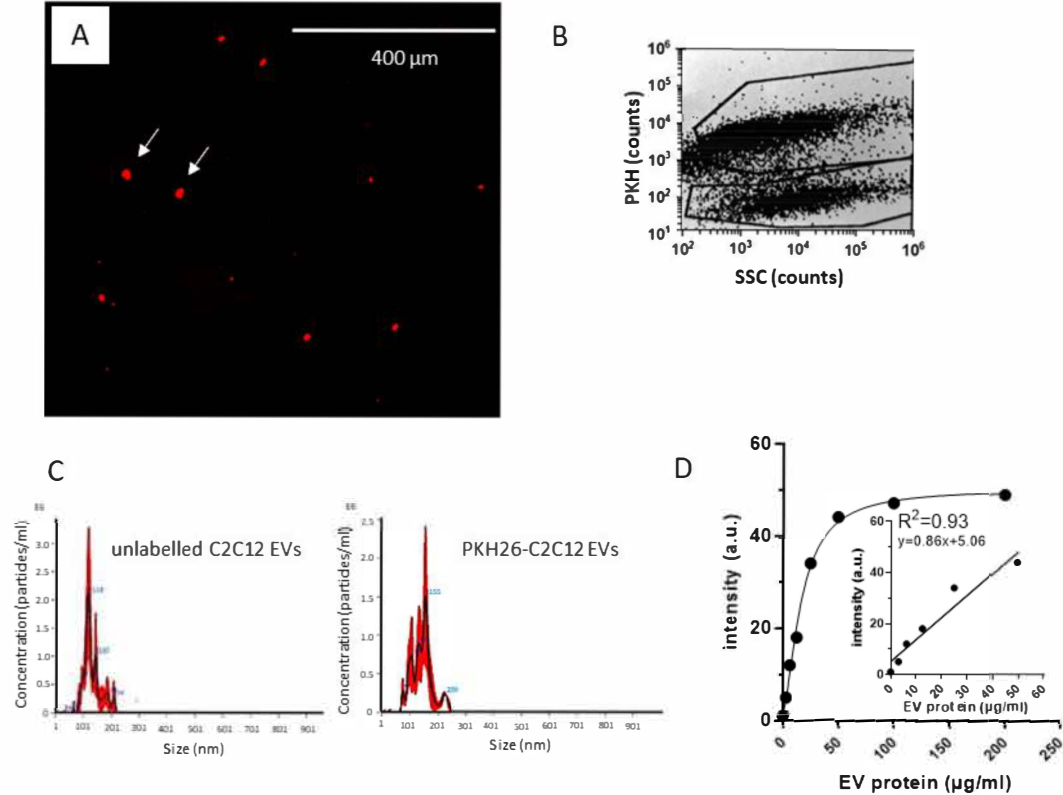

Supplement: Supplementary file 1 [file biology-14-01578-s001.zip › Supplementary Figures f.pdf]
